# Comparative Genomic Analysis of the Regulation of Aromatic Metabolism in Betaproteobacteria

**DOI:** 10.3389/fmicb.2019.00642

**Published:** 2019-03-29

**Authors:** Inna A. Suvorova, Mikhail S. Gelfand

**Affiliations:** ^1^Institute for Information Transmission Problems RAS (The Kharkevich Institute), Moscow, Russia; ^2^Faculty of Computer Science, Higher School of Economics, Moscow, Russia; ^3^Center of Life Sciences, Skolkovo Institute of Science and Technology, Moscow, Russia

**Keywords:** comparative genomics, transcription factor binding site (TFBS), aromatic metabolism regulation, benzoate, phenol, biphenyl, catechol, chlorocatechol

## Abstract

Aromatic compounds are a common carbon and energy source for many microorganisms, some of which can even degrade toxic chloroaromatic xenobiotics. This comparative study of aromatic metabolism in 32 Betaproteobacteria species describes the links between several transcription factors (TFs) that control benzoate (BenR, BenM, BoxR, BzdR), catechol (CatR, CatM, BenM), chlorocatechol (ClcR), methylcatechol (MmlR), 2,4-dichlorophenoxyacetate (TfdR, TfdS), phenol (AphS, AphR, AphT), biphenyl (BphS), and toluene (TbuT) metabolism. We characterize the complexity and variability in the organization of aromatic metabolism operons and the structure of regulatory networks that may differ even between closely related species. Generally, the upper parts of pathways, rare pathway variants, and degradative pathways of exotic and complex, in particular, xenobiotic compounds are often controlled by a single TF, while the regulation of more common and/or central parts of the aromatic metabolism may vary widely and often involves several TFs with shared and/or dual, or cascade regulation. The most frequent and at the same time variable connections exist between AphS, AphR, AphT, and BenR. We have identified a novel LysR-family TF that regulates the metabolism of catechol (or some catechol derivative) and either substitutes CatR(M)/BenM, or shares functions with it. We have also predicted several new members of aromatic metabolism regulons, in particular, some COGs regulated by several different TFs.

## Introduction

Aromatic compounds such as phenol, toluene, xylene, benzoate are the second most abundant class of organic compounds after carbohydrates and a common carbon and energy source for many microorganisms (Arai et al., 1999; López Barragán et al., 2004; Gescher et al., 2005; Carmona et al., 2009; Li et al., 2010; Marín et al., 2010; Valderrama et al., 2012). In natural habitats, they are accumulated mainly due to the degradation of plant-derived molecules (e.g., lignin) (Li et al., 2010). On the other hand, stability of the benzene ring together with massive production and release of various xenobiotic aromatic compounds into the environment makes them one of the most significant classes of pollutants (López Barragán et al., 2004; Carmona et al., 2009). Notably, some bacteria can grow on toxic chloroaromatic compounds, e.g., (poly)chlorinated phenol or biphenyl derivatives, which are serious environmental pollutants generated by human industrial activities (Ohtsubo et al., 2001; Chávez et al., 2004; Takeda et al., 2004). For example, soil bacteria can degrade 2,4-dichlorophenoxyacetic acid (2,4-D), a widely used herbicide (Vedler et al., 2004; Trefault et al., 2009). Thus, bacterial metabolism of aromatic compounds and its regulation is an important research object.

Structurally diverse aromatic compounds are usually converted to a limited number of simpler common intermediates, such as benzoate or catechol, which are subsequently channeled into the tricarboxylic acid (TCA) cycle (Cowles et al., 2000; Tover et al., 2000, 2001; Carmona et al., 2009; Li et al., 2010; Marín et al., 2010). Hence, aromatic biodegradative routes may be divided into the *upper* and *lower* pathways, the latter connected with the few central intermediates, and combinations of various *upper* pathways with standard *lower* pathways can sufficiently broaden substrate specificities of bacteria and are an important factor in their adaptation to xenobiotics in the environment (Klemba et al., 2000).

Genes of various aromatic metabolic pathways, especially those involved in the degradation of xenobiotic compounds, are often located on plasmids, thus allowing for their efficient lateral transfer and adaptation of bacteria to novel compounds, expanding their catabolic abilities (Arai et al., 1999; Klemba et al., 2000; Vedler et al., 2004). Moreover, sets of aromatic metabolism genes are often organized in large supraoperonic clusters and associated with mobile DNA elements, in particular ICE (integrative and conjugative elements, characterized by insertion into tRNA^Gly^genes), that are likely responsible for their transfer and insertion (Laemmli et al., 2000; Carmona et al., 2009; Lechner et al., 2009; Zamarro et al., 2016; Miyazaki et al., 2018).

While various aromatic metabolic pathways, their biochemistry and regulation are rather well-studied in many bacteria, not much is known about connections and interactions between the regulators of these pathways. Here we describe the links between several transcription factors (TFs) that control different steps of aromatic metabolism (including dual and cascade regulation) and demonstrate variability in the organization of the aromatic metabolism operons and the structure of the regulatory networks even between closely related species.

### Phenol, Biphenyl, and Toluene Metabolism

The *aph* and *bph* genes encode proteins of the phenol and biphenyl (or polychlorinated biphenyl) degradation, respectively (Arai et al., 1999, 2000; Ohtsubo et al., 2001; Takeda et al., 2004).

**FIGURE 1:**
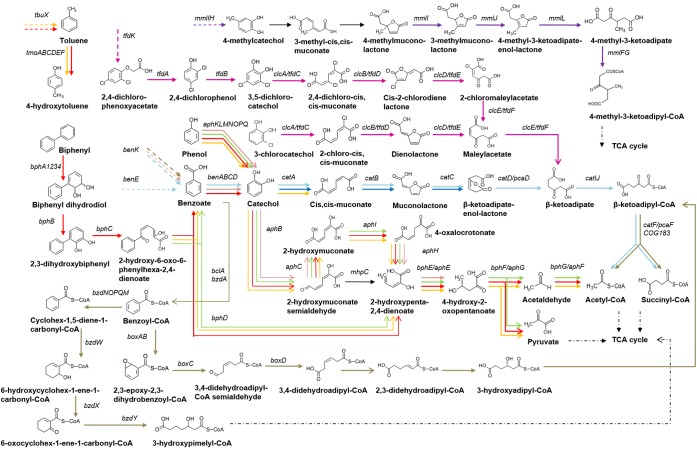
Scheme of benzoate, phenol, biphenyl, toluene, 2,4-dichlorophenoxyacetate, 3-chlorocatechol and 4-methylcatechol utilization and its regulation in Betaproteobacteria. Dashed arrows denote transporters, arrow color denotes regulation by the following TFs: AphS/BphS – red, AphR – orange, AphT – green, BenR – pink, BoxR/BzdR – brown, CatR and/or CatM/BenM – light blue, CatR^∗^ – dark blue, ClcR and TfdR(S) – magenta.

In the *aph*-encoded pathway, phenol is converted into catechol via multi-component phenol hydroxylase AphKLMNOP (and ferredoxin AphQ), which further goes through the *meta*-cleavage by the catechol-2,3-dioxygenase AphB (Arai et al., 1999, 2000). Resulting 2-hydroxymuconate semialdehyde can be converted yielding 2-hydroxypenta-2,4-dienoate and formate either directly by a 2-hydroxymuconic semialdehyde hydrolase (COG596, MhpC/DmpD/XylF) or through 2-hydroxymuconate and 4-oxalocrotonate via the 2-hydroxymuconic semialdehyde dehydrogenase AphC, 4-oxalocrotonate tautomerase AphI, and 4-oxalocrotonate decarboxylase AphH (Figure 1 and Supplementary Table S1) (Arai et al., 2000).

Biphenyl is converted via the biphenyl 2,3-dioxygenase BphA into biphenyl dihydrodiol (2,3-dihydroxy-4-phenylhexa-4,6-diene), which is then transformed into 2,3-dihydroxybiphenyl by the 2,3-dihydrobiphenyl-2,3-diol dehydrogenase BphB (Ohtsubo et al., 2001; Takeda et al., 2004). The 2,3-dihydroxybiphenyl ring is cleaved by the 2,3-dihydroxybiphenyl-1,2-dioxygenase BphC, and the resulting intermediate (2-hydroxy-6-oxo-6-phenylhexa-2,4-dienoate) is split into benzoate and 2-hydroxypenta-2,4-dienoate by the 2-hydroxy-6-oxo-6-phenylhexa-2,4-dienoate hydrolase BphD (Ohtsubo et al., 2001; Takeda et al., 2004).

Further reactions are common for the phenol and biphenyl utilization pathways: 2-hydroxypenta-2,4-dienoate is converted to acetyl-CoA and pyruvate by the consecutive action of the 2-hydroxypenta-2,4-dienoate hydratase AphE/BphE, 4-hydroxy-2-ketovalerate aldolase AphG/BphF, and acetaldehyde dehydrogenase AphF/BphG (Figure 1 and Supplementary Table S1) (Arai et al., 2000; Ohtsubo et al., 2001; Takeda et al., 2004).

Similarly to reactions with phenol, toluene can be converted via multi-component toluene-2-monoxygenase (*tbmABCDEF*, e.g., in *Burkholderia vietnamiensis* G4), toluene-3-monooxygenase (*tbuA1UBVA2C*, e.g., in *Ralstonia pickettii* PKO1) or toluene-4-monooxygenase (*tmoABCDE*, e.g., in *Pseudomonas mendocina* KR1) to *o*-cresol, *m*-cresol, or *p*-cresol, respectively, which are subsequently metabolized through *ortho*- or *meta*-cleavage pathways (Yen et al., 1991; Byrne et al., 1995; Byrne and Olsen, 1996; Leahy et al., 1997; Kahng et al., 2000).

In general, the *aph* and *bph* genes are organized into long operons, and their expression is controlled by one or more TFs, usually transcribed divergently from the regulated genes (Arai et al., 1999, 2000; Ohtsubo et al., 2001; Teramoto et al., 2001; Takeda et al., 2004). Regulators of the aromatic catabolism are usually activators, though there are examples of repressor-mediated regulation (Ohtsubo et al., 2001). For example, in *Comamonas testosteroni* TA441, the *aph* gene cluster is regulated by the GntR-family repressor AphS, activator AphR from the XylR/DmpR subclass of the NtrC family (TFs from this group often control multi-component phenol hydroxylases), and the LysR-family activator AphT (CatR/ClcR-type) (Arai et al., 1999, 2000; Teramoto et al., 2001). AphS and AphR control expression of their own *aphRS* operon and divergently transcribed genes of phenol hydroxylase and catechol-2,3-dioxygenase, while other *aph* genes of the lower pathway are under dual regulation by AphR and AphT (Arai et al., 1999, 2000; Teramoto et al., 2001). Toluene metabolism genes in some bacteria also are regulated by homologous TFs from the XylR/DmpR group (e.g., TbuT in *R. pickettii* PKO1, TbmR in *B. vietnamiensis* G4) (Byrne and Olsen, 1996; Leahy et al., 1997; Kahng et al., 2000).

AphS is highly similar to the repressor of the *bph* genes, BphS, described in, e.g., *Burkholderia xenovorans* LB400 and *Ralstonia eutropha* A5 (not to be confused with BphS from the BphST two-component system, e.g., in *Rhodococcus* sp. RHA1) (Arai et al., 1999; Ohtsubo et al., 2001; Takeda et al., 2004). The binding motif of AphS and BphS is a palindrome with the consensus AwATATCGATATwT; multiple sites have been identified upstream of the regulated genes, which implies cooperative TF binding (Arai et al., 1999; Ohtsubo et al., 2001). The putative AphR binding motif, GCTTGATCATTTGATCATGC, predicted in *Comamonas testosteroni* TA441, is similar to the recognition sites of DmpR, XylR, and HbpR that are TFs from the same family (Arai et al., 1999; Jaspers et al., 2001; Tropel and van der Meer, 2002).

### Benzoate and (Chloro/Methyl)Catechol Metabolism

Bacteria employ three different strategies for the degradation of benzoate – aerobic, anaerobic, and the hybrid one, which has features of both aerobic and anaerobic pathways (Valderrama et al., 2012; Chen et al., 2014). The classical aerobic pathway starts with hydroxylation of benzoate by mono- and dioxygenases into catechol, protocatechuate, or gentisate, e.g., in a two-step process catalyzed by BenABC and BenD (Collier et al., 1998; Cowles et al., 2000; Gescher et al., 2005, 2006; Li et al., 2010; Chen et al., 2014). These compounds are further cleaved by various dioxygenases either between the two hydroxyl groups (*ortho*-cleavage, β-ketoadipate pathway) or next to one of the hydroxyls (*meta*-cleavage) (Collier et al., 1998; Cowles et al., 2000; Gescher et al., 2002, 2005; Li et al., 2010; Chen et al., 2014). The *ortho*-cleavage pathways of catechol, 3-chlorocatechol, and 2,4-D (encoded by the *cat*, *clc*, and *tfd* genes, respectively) are parallel catabolic pathways that convert these compounds into the common intermediate β-ketoadipate, and further channel it into the TCA cycle, namely into acetyl-CoA and succinyl-CoA (Parsek et al., 1994; McFall et al., 1997b; Chugani et al., 1998; Laemmli et al., 2000; Vedler et al., 2004; Ezezika et al., 2006; Li et al., 2010). This conversion involves aromatic ring cleavage into (chloro)muconate and formation of corresponding lactones, carried out by homologous enzymes – dioxygenases CatA, ClcA, and TfdC, and cycloisomerases CatB, ClcB, and TfdD, respectively (Figure 1 and Supplementary Table S1) (McFall et al., 1997a,b; Chugani et al., 1998; Tover et al., 2001).

The pathways mentioned above are regulated by homologous TFs from the LysR family: the *cat* genes are controlled by CatR or CatM (e.g., in *Pseudomonas putida* and *Acinetobacter baylyi* ADP1, respectively); the *clc* genes, by ClcR (e.g., in *P. putida*); and the *tfd* genes (e.g., in *Ralstonia eutropha* JMP134), by TfdR and an almost identical paralog, TfdS (Parsek et al., 1994; Leveau and van der Meer, 1996; Chugani et al., 1997, 1998; McFall et al., 1997a,b; Collier et al., 1998; Laemmli et al., 2000; Tover et al., 2000, 2001; Ezezika et al., 2006). Moreover, they are not only homologous, but share functions and can partially substitute each other (Parsek et al., 1994; McFall et al., 1997b; Trefault et al., 2009). The genes of all these LysR-family TFs are divergently transcribed from the regulated operons (Parsek et al., 1994; Leveau and van der Meer, 1996; Chugani et al., 1997, 1998; McFall et al., 1997a,b; Laemmli et al., 2000). The binding motifs of these TFs are similar inverted repeats with the consensus AkACC–N_5_–GGTAT, conforming to the general LysR-family motif T–N_11_–A (Parsek et al., 1994; Chugani et al., 1997, 1998; McFall et al., 1997a,b; Laemmli et al., 2000; Tover et al., 2000).

Benzoate metabolism is regulated by the AraC/XylS-type activator BenR (e.g., in *Pseudomonas putida*) and LysR-family activator BenM (e.g., in *Acinetobacter baylyi* ADP1) (Collier et al., 1998; Cowles et al., 2000; Ezezika et al., 2006; Li et al., 2010). BenR binding sites are repeated sequences with consensus TGCA–N_6_–GGNTA (Cowles et al., 2000; Li et al., 2010). Highly homologous regulators BenM and CatM bind similar DNA motifs and can partially substitute each other, playing overlapping roles in controlling the expression of the *ben* and *cat* genes, which are involved in the benzoate consumption and catabolism in response to different effectors (Collier et al., 1998; Ezezika et al., 2006; Li et al., 2010).

The first step of the anaerobic benzoate degradation is its activation to benzoyl-CoA by benzoate-CoA ligase BzdA (Geissler et al., 1988; Gescher et al., 2002; López Barragán et al., 2004; Barragán et al., 2005; Durante-Rodríguez et al., 2008; Carmona et al., 2009; Valderrama et al., 2012). The next reaction is reductive dearomatization of the aromatic ring by benzoyl-CoA reductase (BcrCBAD/BadDEFG/BzdNOPQ with ferredoxin Fdx/BadB/BzdM as an electron donor), yielding cyclohex-1,5-diene-1-carbonyl-CoA or cyclohex-1-ene-carbonyl-CoA (Koch and Fuchs, 1992; López Barragán et al., 2004; Durante-Rodríguez et al., 2008; Carmona et al., 2009; Valderrama et al., 2012; Chen et al., 2014).

Through a series of reactions including hydrolytic ring cleavage (carried out by the hydratase Dch/BadK/BzdW, dehydrogenase Had/BadH/BzdX, hydrolase Oah/BadI/BzdY), these intermediates are subsequently converted into 3-hydroxypimelyl-CoA or pimelyl-CoA, and finally converted via β-oxidation-like reactions and decarboxylation into acetyl-CoA, which is channeled into the central metabolism (Figure 1 and Supplementary Table S1) (Carmona et al., 2009; López Barragán et al., 2004; Valderrama et al., 2012; Chen et al., 2014).

Another way of the benzoate catabolism is the aerobic hybrid pathway (CoA-dependent epoxide *box* pathway), starting with the activation of benzoate to benzoyl-CoA by benzoate-CoA ligase BclA, as in the anaerobic metabolism (Gescher et al., 2002; Valderrama et al., 2012; Chen et al., 2014). Some bacteria, for example, *Thauera* and *Magnetospirillum* strains, have one benzoate-CoA ligase for both *bzd* and *box* pathways and a single regulator, while others, e.g. *Azoarcus* sp. CIB and *Azoarcus aromaticum* EbN1 strains, have two different ligases and regulators, for the aerobic (BclA and BoxR) and anaerobic (BzdA and BzdR) pathways, respectively (Carmona et al., 2009; Valderrama et al., 2012). Benzoyl-CoA oxygenase BoxAB and dihydrolase BoxC subsequently oxidatively dearomatize and hydrolytically cleave benzoyl-CoA into 3,4-dehydroadipyl-CoA semialdehyde (Gescher et al., 2002, 2005, 2006; Valderrama et al., 2012; Chen et al., 2014). The latter is oxidized by 3,4-dehydroadipyl-CoA semialdehyde dehydrogenase BoxD to 3,4-dehydroadipyl-CoA (Gescher et al., 2006; Chen et al., 2014), which is further converted through several coenzyme A thioesters to succinyl-CoA and acetyl-CoA via reactions similar to the β-oxidation and β-ketoadipate pathway (Figure 1 and Supplementary Table S1) (Gescher et al., 2002, 2006; Valderrama et al., 2012; Chen et al., 2014). Both aerobic and anaerobic metabolic pathways for the benzoate degradation may occur simultaneously (Valderrama et al., 2012; Chen et al., 2014). The *box* pathway is more widely distributed among Betaproteobacteria than in the other taxa (Chen et al., 2014).

BoxR and BzdR are closely related repressor proteins that have an N-terminal DNA-binding domain homologous to that of xenobiotic response element (XRE) transcriptional regulators and a C-terminal domain similar to shikimate kinase (PF01202) (Barragán et al., 2005; Durante-Rodríguez et al., 2008, 2010; Valderrama et al., 2012). BzdR controls expression of the adjacent *bzd* genes involved in the anaerobic degradation of benzoate; the genes are typically organized in a single operon, e.g., *bzdNOPQMSTUVWXYZA* in *Azoarcus* sp. CIB (López Barragán et al., 2004; Barragán et al., 2005; Durante-Rodríguez et al., 2008, 2010; Valderrama et al., 2012). BoxR represses the aerobic hybrid pathway of the benzoate utilization encoded by the *box* genes (Valderrama et al., 2012).

The BoxR and BzdR regulators are highly similar not only in sequence, but also in the DNA recognition mechanism, they act synergistically and cross-regulate the *box* and *bzd* genes, possibly as an adaptation to changing oxygen concentrations (Carmona et al., 2009; Valderrama et al., 2012). Both BoxR and BzdR bind promoter regions containing several repeats of the TGCA sequence that usually form longer palindromes, possibly cooperatively binding several BzdR dimmers (Barragán et al., 2005; Durante-Rodríguez et al., 2008, 2010; Valderrama et al., 2012). This conforms to the fact that other TFs from the HTH-XRE family also bind short repeated sequences that, in most cases, are located within palindromic regions (Barragán et al., 2005; Durante-Rodríguez et al., 2008). These TGCA sequences are separated by 1, 6, or 15 nucleotides (Barragán et al., 2005; Valderrama et al., 2012).

Degradation of 4-methylcatechol via *ortho*-cleavage usually leads to the formation of 4-methylmuconolactone, which bacteria are rarely able to utilize (Marín et al., 2010). The process involves 4-methylmuconolactone isomerization and rearrangement of the double bond by methylmuconolactone isomerases MmlI and MmlJ, while resulting 4-methyl-3-ketoadipate enol-lactone is converted into 4-methyl-3-ketoadipate via 4-methyl-3-ketoadipate enol-lactone hydrolase MmlL (Marín et al., 2010). Further metabolism is analogous to the reactions of the β-ketoadipate pathway: 4-methyl-3-ketoadipyl-CoA-transferase MmlFG and thiolase MmlC consequently transform 4-methyl-3-ketoadipate into methylsuccinyl-CoA and acetyl-CoA (Figure 1 and Supplementary Table S1) (Marín et al., 2010). The *mml* operon includes *mmlR* that encodes a LysR-family TF likely regulating the operon transcription (Marín et al., 2010).

### Goals

Here, we apply comparative genomics methods to characterize the regulation of the main aromatic metabolic pathways in a representative set of Betaproteobacteria. We reconstruct corresponding regulons and predict new regulon members and their function, and describe the interactions between the studied TFs.

## Materials and Methods

For the comparative study we selected 32 genomes available for the analysis with RegPredict (Novichkov et al., 2010), which we consider to be a relatively unbiased, representative set of Betaproteobacteria (Supplementary Table S2). The genomes were downloaded from GenBank (Benson et al., 1999).

Homologs of proteins were identified by PSI-BLAST (Altschul et al., 1997) (*E*-value cutoff, 10^-20^), and orthologs were determined by construction of phylogenetic trees and analysis of the genomic context (e.g., co-localization with genes of a certain metabolic pathway in most genomes). Amino acid and nucleotide sequence alignments were performed using the MUSCLE tool (default parameters) (Edgar, 2004). Phylogenetic trees were built using PhyML (default parameters) (Dereeper et al., 2008) and visualized with Dendroscope (Huson et al., 2007).

Candidate binding sites were identified (or confirmed if they had been previously predicted) by phylogenetic footprinting, that is, manual inspection of alignments of orthologous upstream regions with identification of consecutive conserved nucleotide positions, following the assumption that binding sites are more conserved than intergenic regions in general (Rodionov, 2007). Nucleotide position weight matrices (PWMs) for each TF were constructed by the SignalX program as previously described (Gelfand et al., 2000), using training sets of upstream regions of genes presumably belonging to the respective regulons. PWMs were then used to search for additional regulon members.

Computational search for candidate binding sites in upstream gene regions [350 nucleotides (nt) upstream and 50 nt downstream relative to the annotated gene start] was performed using the GenomeExplorer program package (Mironov et al., 2000). Score thresholds for the identification of sites were selected so that candidate sites upstream of functionally relevant genes were accepted, while the fraction of genes preceded by candidate sites did not exceed 5% in each studied genome. Weaker sites (with scores 10% less than the threshold) were also taken into account if their positions were similar to positions of stronger sites upstream of orthologous genes and/or there were no stronger competing sites in the same intergenic region. New candidate members were assigned to a regulon if they were preceded by candidate binding sites in several genomes. The reconstructed regulons were extended to include all genes in putative operons, the latter defined as the strings of genes transcribed in the same direction, with intergenic distances not exceeding 200 nt, when such organization persisted in several genomes. Motif logos were constructed using WebLogo (Crooks et al., 2004).

## Results

Metabolic pathways of aromatic compounds are diverse, and their regulation is often complex and evolutionally labile. Here we analyze the utilization pathways of phenol, biphenyl, benzoate, catechol and its chloro- and methyl-derivatives, describe their regulation in Betaproteobacteria, reconstruct the regulons, identify new regulon members, and predict their metabolic functions.

### Regulation of the Phenol, Biphenyl, Toluene and Benzoate Metabolism by AphS/BphS, AphR, AphT, and BenR

AphS and BphS are very close homologs and their binding motifs have a similar structure. Hence, here these TFs have been assigned names AphS or BphS according to the branching of the phylogenetic tree (Supplementary Figure S1) and co-localization of the regulatory genes with either phenol or biphenol metabolic genes (genomic context).

**FIGURE 2:**
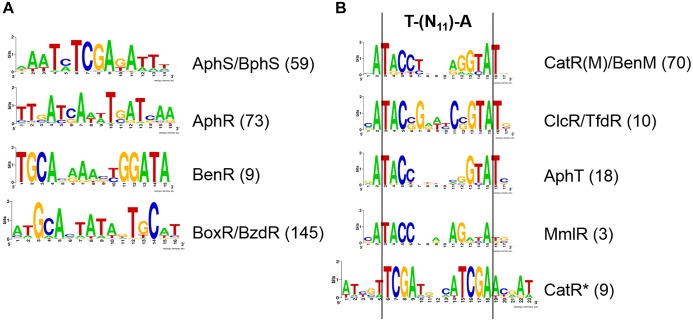
LOGO diagrams of transcription factor binding motifs. **(A)** AphS/BphS, AphR, BenR, and BoxR/BzdR. **(B)** LysR-family regulators of the aromatic metabolism. The LOGO diagrams were built using all the predicted binding sites for the respective TF in all studied genomes (see Supplementary Table S3), the exact numbers of sites are given in parentheses.

Phylogenetic footprinting confirmed, and further site search refined the previously described AphS/BphS binding motif (Ohtsubo et al., 2001), which is an even 14 nt palindrome with the consensus AAATmTCGAkATTT (Figure 2A).

**FIGURE 3:**
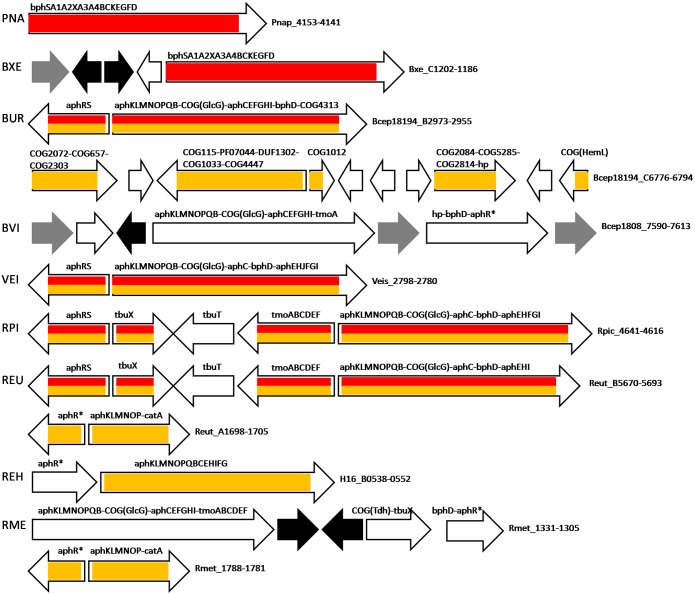
Operon organization and regulation of aromatic metabolism genes regulated by AphS/BphS and AphR in *Burkholderia* spp., *P. naphthalenivorans* CJ2, *Ralstonia* spp., and *V. eiseniae* EF01-2. Color denotes regulation by the following TFs: AphS/BphS – red, AphR – orange. Double coloring denotes dual regulation. Genes/operons are shown by arrows, black arrows denote pseudogenes, gray – transposases; “hp” indicates hypothetical proteins. Genome abbreviations as in Supplementary Table S2.

Among the studied bacteria, BphS was identified only in *Burkholderia xenovorans* LB400 and *Polaromonas naphthalenivorans* CJ2, which also lack AphR, AphT, and *aph* genes of phenol utilization, and in *Azoarcus* sp. BH72 (see below). The BphS regulon in *B. xenovorans* LB400 and *P. naphthalenivorans* CJ2 consists of one operon, *bphSA1A2XA3A4BCKEGFD*, that is comprised of the regulatory gene and genes encoding the complete pathway of the biphenyl conversion into pyruvate and acetyl-CoA (Figure 3 and Supplementary Tables S1, S2). Close homologs (possibly orthologs) of some *bph* genes are identified in *B. petrii* DSM 12804, which has neither AphS/BphS, AphR, nor AphT; they are clustered together with the *clc* genes, but are not regulated by either ClcR or CatR, and may be under regulation of an adjacent AraC-family TF (*Bpet3746*).

Interactions between regulators AphS, AphR, AphT, and BenR, and the operon organization of their regulated genes vary, and the exposition below is structured according to these interactions, starting with bacteria that have only AphS and AphR TFs as the regulators of the phenol metabolism, and continued with bacteria where the phenol metabolism is regulated by AphS, AphR, and AphT.

Bacteria having AphS always have AphR as well, their genes usually forming the *aphRS* operon. *Burkholderia* sp. 383, *Ralstonia eutropha* JMP134, *Ralstonia pickettii* 12J, and *Verminephrobacter eiseniae* EF01-2 have only AphS and AphR as the phenol regulators, and their *aph* metabolic genes are organized in a long operon under dual regulation (Figure 3 and Supplementary Tables S2, S3).

Moreover, in *R. eutropha* JMP134 and *R. pickettii* 12J, the toluene transport and metabolic genes *tbuX* (Kahng et al., 2000; van den Berg et al., 2015) and *tmoABCDEF*, localized between *aphRS* and other *aph* genes, are regulated not only by their own repressor TbuT, but by both AphS and AphR. In *Ralstonia metallidurans* CH34 that lacks AphS, AphR, and TbuT, the *tmoABCDEF* genes are likely co-transcribed with the *aph* genes as a single operon, possibly under regulation of the AphR/TbuT homolog (*Rmet_1305*, Supplementary Figure S2), though no binding motif of this TF could be identified. *Burkholderia vietnamiensis* G4 features the same organization of this operon, except having only *tmoA* out of all toluene metabolic genes, possibly as a result of disruption of the *tmo* gene cluster by transposases. This operon is also likely regulated by an AphR/TbuT homolog, though no AphR-type binding site has been found (Figure 3, Supplementary Figure S2, and Supplementary Table S2). In *Dechloromonas aromatica* RCB, the *tmoABCDEF–tbuX* operon is regulated by AphS and is localized in a large locus of aromatic metabolism genes, close to an operon controlled by AphT (see below).

**FIGURE 4:**
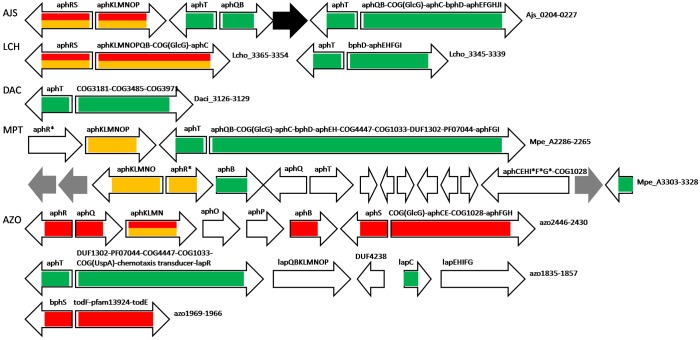
Operon organization and regulation of aromatic metabolism genes regulated by AphS/BphS, AphR, and AphT in other Comamonadaceae and *Azoarcus* sp. BH72. Color denotes regulation by the following TFs: AphS/BphS – red, AphR – orange, AphT – green. Double coloring denotes dual regulation. Genes/operons are shown by arrows, black arrows denote pseudogenes, gray – transposases. Genome abbreviations as in Supplementary Table S2.

Most *aph* genes in *Azoarcus* sp. BH72 are predicted to be regulated by AphS (*azo2437*), except *aphKLMN* that is under dual control by AphS and AphR; BphS in this species (*azo1969*) likely regulates its own expression and the divergent operon, comprised of homologs of *bphD* and *bphC*, and a pfam13924 hypothetical protein with a lipocalin-like domain (Figure 4 and Supplementary Table S2).

All metabolic *aph* genes in *R. eutropha* H16, as well as the second copy of *aphKLMNOP* genes with *catA* (whose orthologs are generally regulated by CatR(M)/BenM) in *R. eutropha* JMP134 and *R. metallidurans* CH34 are presumably regulated by AphR/TbuT homologs with the binding sites similar to the AphR motif (Figure 3, Supplementary Figure S2, and Supplementary Tables S2, S3).

Moreover, in *Burkholderia* sp. 383, AphR likely also controls several adjacent operons formed by COG2072 flavin-containing monooxygenase, COG657 esterase/lipase, COG2303 dehydrogenase possibly associated with lipid metabolism; COG115 aminotransferase or putative 4-amino-4-deoxychorismate lyase (yielding 4-aminobenzoate), a DUF1302 protein, PF07044 and COG1033 exporters, and COG4447 glycosyl hydrolase (their possible role in aromatic metabolism discussed further); COG1012 aldehyde dehydrogenase; COG2084 3-hydroxyacid dehydrogenase, COG5285 phytanoyl-CoA dioxygenase, COG2814 efflux permease and hypothetical protein; COG1 (HemL) aminotransferase/aminomutase (Figure 3 and Supplementary Tables S2, S3).

**FIGURE 5:**
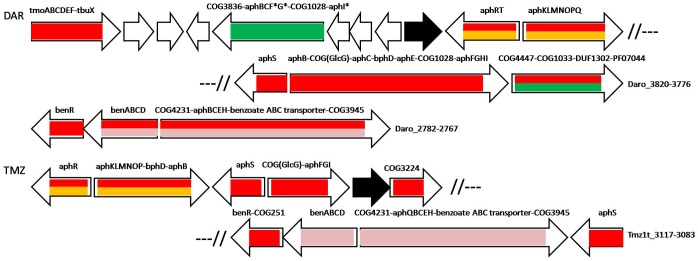
Operon organization and regulation of aromatic metabolism genes regulated by AphS/BphS, AphR, AphT and BenR in *D. aromatica* RCB and *Thauera* sp. MZ1T. Color denotes regulation by the following TFs: AphS/BphS – red, AphR – orange, AphT – green, BenR – pink. Double coloring denotes dual regulation. Genes/operons are shown by arrows, black arrows denote pseudogenes. Operons flanked with double slashes belong to the same locus. Genome abbreviations as in Supplementary Table S2.

AphT was identified only in several Commamonadaceae and Rhodocyclales (Figure 4, 5 and Supplementary Table S2). The predicted AphT binding motif is an odd palindrome that conforms to the general LysR-family consensus (T–N_11_–A) and is similar to the CatR and ClcR motifs (Figure 2B).

In some bacteria, AphT complements AphS and AphR in regulation of the *aph* genes. AphS, AphR and its homologs always regulate genes of phenol hydroxylase subunits, while other *aph* metabolic genes can be controlled by AphT (Supplementary Table S2). Notably, in *Leptothrix cholodnii* SP-6, the *aph* genes that are controlled by AphT and those under dual AphS and AphR regulation are in different loci, while in *Acidovorax* sp. JS42, all *aph* genes, including *aphR*, *aphS*, and two copies of *aphT*, are in the same locus, forming three adjacent pairs of divergent operons (Figure 4 and Supplementary Table S2). In *Methylibium petroleiphilum* PM1 that has neither AphS, nor AphR, the regulation is similarly divided between AphT and an AphR/TbuT homolog (Supplementary Figure S2), genes forming four operons in a single locus (*Mpe_A2286–2265*). The second cluster of the *aph* genes in *M. petroleiphilum* PM1 is also organized in several operons, some of which are regulated by another AphR/TbuT homolog (Supplementary Figure S2) or by a second copy of AphT. This AphT paralog was possibly earlier involved in the control of *aphCEH*, and, as in *Dechloromonas aromatica* RCB, of the close homologs (but not orthologs, data not shown) of *aphIFG*, and of a COG1028 gene. But the operon has been disrupted by a transposase, thus only the first gene, encoding 5-carboxymethyl-2-hydroxymuconate semialdehyde dehydrogenase (*Mpe_A3328*), that is preceded by a strong AphT-binding site, is currently under its regulation (Figure 4 and Supplementary Tables S2, S3).

In *Delftia acidovorans* SPH-1, that has no *aph* metabolic genes, AphT is predicted to regulate its own gene and a divergent operon comprised of a COG3181 transporter (only weakly similar to COG3181 proteins in other studied aromatic regulons, data not shown), a COG3485 enzyme related to catechol 1,2-dioxygenases, but likely encoding dioxygenase of some catechol derivative, such as 3,4-dihydroxybenzoate or 3,4-dihydroxyphenylacetate, and COG3971 2-oxo-hepta-3-ene-1,7-dioic acid hydratase, an enzyme of the lower 3,4-dihydroxyphenylacetate utilization pathway (Figure 4 and Supplementary Tables S2, S3). Thus, this TF likely regulates the 3,4-dihydroxyphenylacetate utilization.

AphT in *D. aromatica* RCB controls two operons in a large aromatic metabolism gene cluster (Figure 5 and Supplementary Table S2). One of them consists of COG3836 2,4-dihydroxyhept-2-ene-1,7-dioic acid aldolase that is an enzyme of the lower 3,4-dihydroxyphenylacetate utilization pathway, one of three copies of *aphB* and *aphC*, close homologs (but not orthologs according to the phylogenetic tree, data not shown) of *aphF*, *aphG*, and *aphI*, and, finally, COG1028 3-ketoacyl-ACP reductase that may perform some intermediate reaction of the pathway. The second operon, which is also controlled by AphS, is comprised of COG4447 glycosyl hydrolase, COG1033 and PF07044 exporters, and a DUF1302 protein; in *Methylibium petroleiphilum* PM1 these four genes, together with the co-transcribed *aph* genes, are also regulated by AphT (Figure 4 and Supplementary Table S2). A similar composition was observed in *Azoarcus* sp. BH72, where AphT regulates itself and the divergent operon comprised of distant homologs of the four aforementioned genes, as well as genes encoding COG589 universal stress protein, methyl-accepting chemotaxis transducer, and regulator LapR (Figure 4 and Supplementary Table S2). These proteins are possibly involved in sensing some aromatic compounds (as it has been shown that several methyl-accepting chemotaxis proteins are induced while growing on aromatic substrates), coping with their toxic concentrations, and solvent stress response (Wöhlbrand et al., 2008; Carmona et al., 2009; Büsing et al., 2015). LapR, a close homolog of AphR, has been described as an alkylphenol metabolism regulator (Jeong et al., 2003), hence it should control transcription of the adjacent *lap* genes of the alkylphenol metabolism (close homologs of *aph*), forming a regulatory cascade AphT–LapR, but no candidate LapR (AphR-type) binding sites have been identified upstream of the *lap* genes.

AraC-family regulator BenR was found only in two Rhodocyclales, *D. aromatica* RCB and *Thauera* sp. MZ1T. Therefore, to build a reliable PWM we also used predicted BenR binding sites from five species of *Pseudomonas* spp., Gammaproteobacteria (*P. aeruginosa*, *P. entomophila*, *P. fluorescens*, *P. putida*, *P. stutzeri*; data not shown). In *D. aromatica* RCB and *Thauera* sp. MZ1T, BenR controls not only benzoate metabolic and transport *ben* genes, but also complements AphS and AphR in the regulation of several *aph* phenol metabolic genes. Moreover, *benR* is directly regulated by AphS; in *Thauera* sp. MZ1T, in an operon with COG251 enamine deaminase gene. In both these bacteria, BenR regulates two divergent operons: *benABCD* and an operon containing COG4231 2-ketoacid ferredoxin oxidoreductase (distantly homologous to the known phenyl- or other arylpyruvate ferredoxin oxidoreductases), *aphQ* (only in *Thauera* sp. MZ1T), *aphBCEH*, benzoate ABC transporter, and the gene encoding a COG3945 protein (whose possible role is described below in the BoxR section). In *D. aromatica* RCB, this divergon is also regulated by AphS. Other *aph* genes (co-localized with BenR-regulated genes in *Thauera* sp. MZ1T and located in the different locus in *D. aromatica* RCB) are regulated by AphS either with or without AphR, as well as by AphT in *D. aromatica* RCB (Figure 5 and Supplementary Tables S2, S3). Among the studied genomes, these two bacteria have the most complex interaction of aromatic regulators, where AphS can share regulation of metabolic genes with AphR, AphT, and BenR, while simultaneously directly controlling expression of these TFs.

### Regulation of the Benzoate Metabolism by BoxR and BzdR

Among the *Bordetella* spp. studied here, *boxR* and other *box* genes, as well as most other aromatic metabolism genes, are found only in *Bordetella petrii* DSM 12804. Among *Burkholderia* spp., *boxR* and other *box* genes are present in *Burkholderia phymatum* STM815 and *Burkholderia xenovorans* LB400; the latter has two paralogs of *boxR* and *box* genes, apparently resulting from a recent duplication (e.g., see the BoxR phylogenetic tree, Supplementary Figure S3). *Cupriavidus taiwanensis* and *Ralstonia eutropha* H16 also have two copies of *boxR* and metabolic *box* genes, but in these cases, according to the phylogenetic tree, it is more likely a result of horizontal gene transfer. Only *Azoarcus* sp. EbN1 among the studied bacteria has both *boxR* and *bzdR*, named according to their co-localization with the regulated genes: one of these paralogous TFs is co-localized with *box* genes, while the other one is localized within the anaerobic benzoate metabolism gene cluster *bzdR–*hypothetical_protein*–bzdNOPQMSTUVWXYZA–*benzoate*_*ABC_transporter*–benK*. Except for benzoate-CoA ligase, which is a common enzyme for both anaerobic and aerobic epoxide pathways, the *bzd* genes are absent in other bacteria from the studied set. *Tmz1t_2954–2945* genes in *Thauera* sp. MZ1T have orthologs in *Thauera aromatica* annotated as *had–oah–dch–bcrCBAD–fdx* (López Barragán et al., 2004), but these genes are rather distantly homologous to the *bzd* genes from *Azoarcus* sp. EbN1, and we have not found any candidate BzdR/BoxR-binding sites upstream of this operon in *Thauera* sp. MZ1T.

**FIGURE 6:**
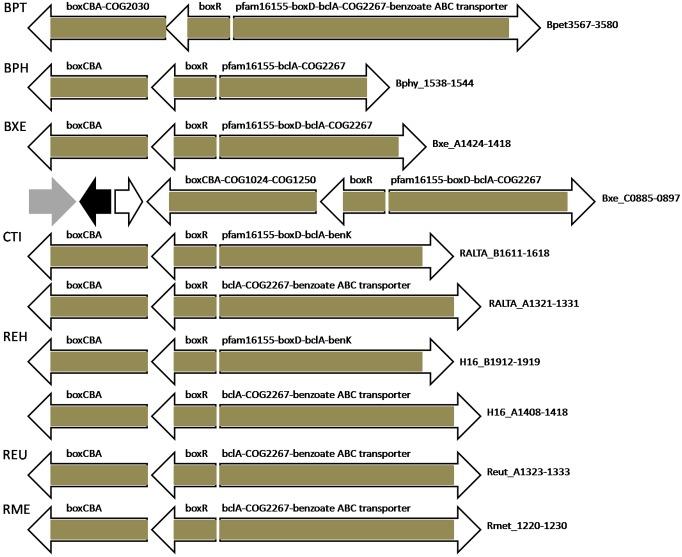
Operon organization and regulation of aromatic metabolism genes regulated by BoxR/BzdR in *B. petrii* DSM 12804, *Burkholderia* spp., and *Ralstonia* spp. Brown color denotes regulation by BoxR/BzdR. Genes/operons are shown by arrows, black arrows denote pseudogenes, gray – transposases. Genome abbreviations as in Supplementary Table S2.

**FIGURE 7:**
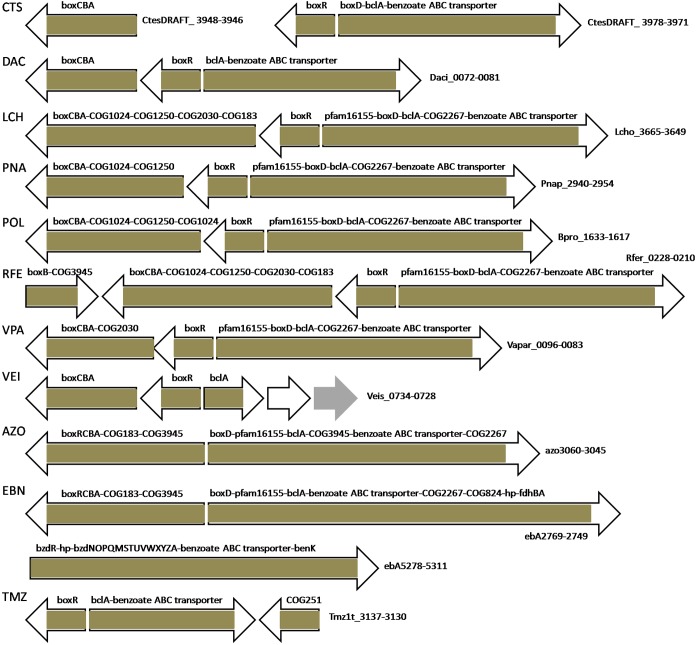
Operon organization and regulation of aromatic metabolism genes regulated by BoxR/BzdR in other studied Betaproteobacteria. Brown color denotes regulation by BoxR/BzdR. Genes/operons are shown by arrows, gray arrows denote transposases; “hp” indicates hypothetical proteins. Genome abbreviations as in Supplementary Table S2.

BoxR-regulated genes in all studied bacteria are organized in divergent clusters, where one part almost always contains separately transcribed (or first in the operon) *boxR* and an adjacent operon including *boxCBA* and, optionally, genes encoding COG1024/COG447, COG1250, COG2030 proteins and COG183 3-ketoadipyl-CoA thiolase (Figure 6, 7 and Supplementary Tables S2, S3). The other part of the divergon is formed by *boxD*, *bclA*, genes encoding COG2267/COG596 lactonase of 3-hydroxyadipyl-CoA lactone (one of additional intermediates of the benzoate utilization via the *box* pathway; Zaar et al., 2001; Gescher et al., 2002), a pfam16155 protein with the cupin-like fold homologous to 4-hydroxylaminobenzoate lyase/2-hydroxylaminobenzoate mutase (likely involved in the metabolism of some benzoate derivative, e.g., protocatechuate; Gescher et al., 2002), and benzoate transporters; some of these genes may be missing and the gene order in an operon may vary depending on the genome (Figure 6, 7 and Supplementary Tables S2, S3).

BoxR regulons in most studied bacteria include putative benzoate ABC transporter genes, whose role in the benzoate uptake has been predicted, but not experimentally demonstrated (Gescher et al., 2002; López Barragán et al., 2004; Carmona et al., 2009; Chen et al., 2014). Notably, in *D. aromatica* RCB, which lacks BoxR, this ABC transporter is located within another benzoate metabolic gene cluster and is under dual regulation by BenR and AphS. Resembling regulation is observed in *Thauera* sp. MZ1T, which has two paralogous copies of this transporter: one is under control of BenR, the other is regulated by BoxR (Figure 5, 7 and Supplementary Table S2). *Polaromonas* sp. JS666 and *Polaromonas naphthalenivorans* CJ2 have two and three paralogous copies of these transporter genes, respectively, where one is a part of the BoxR regulon, while others are likely regulated by IclR-family TFs (not studied here) whose genes are adjacent to the transporter genes. Moreover, in *Cupriavidus taiwanensis* and *Ralstonia eutropha* H16, one copy of *boxR* is associated with this benzoate ABC transporter, while the second one, with *benK*. In *Azoarcus* sp. EbN1 both these transporters are in the same operon with *bzd* genes (Figure 7).

The *box* gene clusters described so far lack genes encoding 3-hydroxyacyl-CoA hydro-lyase and 3-hydroxyadipyl-CoA dehydrogenase to complete the pathway and connect it to the TCA cycle (Gescher et al., 2002). Genes encoding COG1024/COG447 enoyl-CoA hydratase/isomerase and COG1250 3-hydroxyacyl-CoA dehydrogenase in BoxR-regulated operons in *Burkholderia xenovorans* LB400 and in several Comamonadaceae from the selected genome set likely perform these functions (Figure 1).

BoxR regulons of several studied bacteria include a gene encoding COG2030 acyl dehydratase (Figure 6, 7 and Supplementary Table S2). The function of this protein in the benzoate degradation remains unknown, but its sequence is similar to enzymes catalyzing the hydrolytic ring cleavage and aldehyde oxidation during the aerobic phenylacetate metabolism via CoA thioesters (Ferrández et al., 1998; Gescher et al., 2002; Teufel et al., 2011) and enoyl-CoA conversion into 3-hydroxyacyl-CoA (Park and Lee, 2003). Thus, this protein could be involved in the reactions carried out by BoxC and BoxD, or in the further steps of β-oxidation.

The BoxR regulon in *Azoarcus* sp. EbN1 might also include COG824 acyl-CoA thioesterase and formate dehydrogenase FdhBA, since the intergenic distance between these genes and the lactonase gene from the *box* genes is very short and thus they could be co-transcribed. Thioesterase may be required to hydrolyze CoA thioester intermediates of the benzoate degradation pathway if enzymes downstream of benzoyl-CoA become limiting, and thus release CoA preventing its deprivation (Gescher et al., 2002). Formate dehydrogenase may be involved in the oxidation of formate generated by BoxC, providing reducing equivalents (Gescher et al., 2006). In *Azoarcus* sp. BH72, these genes are also localized near the *box* operon, but the intergenic distance is too large to imply co-transcription.

Benzoate metabolic gene clusters (regulated either by BenR and/or AphS, or BoxR, Figure 5, 7 and Supplementary Table S2) in studied Rhodocyclales and *Rhodoferax ferrireducens* DSM 15236 also include genes encoding COG3945 protein with the hemerythrin/HHE cation-binding motif. Its role in the benzoate metabolism is not clear (Gescher et al., 2002), possibly, it could protect the cell from toxic compounds and/or act as an oxygen store/scavenger during the oxygen concentration changes. Indeed, it has been shown that the metabolism of aromatic compounds may cause oxidative stress response (Chávez et al., 2004; Denef et al., 2006; Carmona et al., 2009).

Moreover, we predict that *Tmz1t_3130* encoding enamine deaminase (COG251, YjgF/RidA family) in *Thauera* sp. MZ1T also belongs to the BoxR regulon. Proteins of this family quench reactive enamine/imine intermediates that can inactivate a number of enzymes by covalent binding to their active sites (Lambrecht et al., 2012; Flynn et al., 2013; Flynn and Downs, 2013). In particular, some YjgF-like proteins are known to deaminate short-lived enamines 2-aminomuconate to 4-oxalocrotonate during the degradation of nitrobenzene (He and Spain, 1998; Park and Kim, 2000) and intermediate 2-amino-5-carboxymuconic 6-semialdehyde into 2-hydroxy-5-carboxymuconic 6-semialdehyde in the *meta*-cleavage pathway of 4-amino-3-hydroxybenzoate degradation (Orii et al., 2004; Takenaka et al., 2009). Moreover, chorismatase XanB2 that converts chorismate into 3-hydroxybenzoate and 4-hydroxybenzoate has an YjgF-like domain and is important for the oxidative stress defense (Zhou et al., 2013). Thus, Tmz1t_3130 may indeed play a role in the utilization of a benzoate derivative and stress resistance.

Experimental studies of BoxR and BzdR have demonstrated that they bind TGCA repeats separated by either 1, 6, or 15 nucleotides (Barragán et al., 2005; Durante-Rodríguez et al., 2008, 2010; Valderrama et al., 2012). Here we show that, according to the phylogenetic footprinting data, the main variant of the motif has 6 nt between TGCA, forming the ATGCACTATAGTGCAT palindrome (Figure 2A). The identified sites are almost always multiple, often separated by several DNA turns, mainly 3–5 (Supplementary Table S3).

### Regulation of the Catechol Metabolism by CatR and CatM/BenM

**FIGURE 8:**
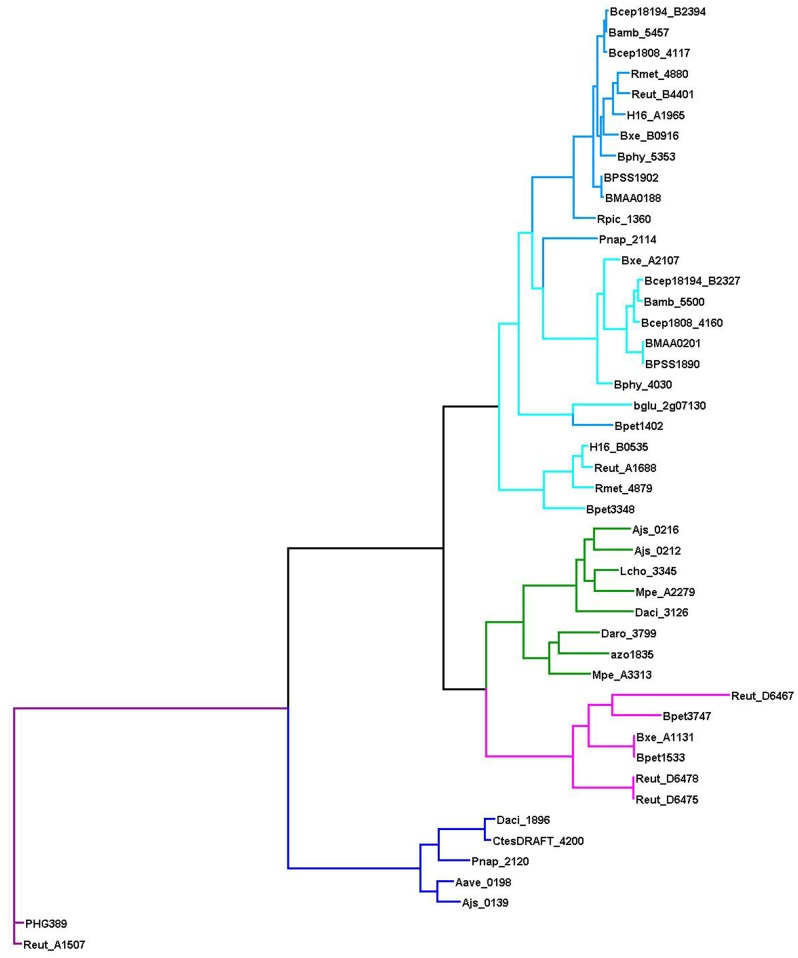
Phylogenetic tree of the LysR-family regulators of the aromatic metabolism. The color code is consistent with that in Figure 1: AphT – green, CatR – light blue, CatM/BenM – blue, CatR^∗^ – dark blue, ClcR and TfdR(S) – magenta, MmlR – violet.

The nomenclature of homologous LysR-family TFs that control the catechol and/or benzoate metabolism is not consistent and depends on the species they are characterized in Chugani et al. (1997), Collier et al. (1998), Cowles et al. (2000), Tover et al. (2001), Clark et al. (2004), Ezezika et al. (2006). CatR and CatM/BenM likely substitute each other, completely or partially, but it can be analyzed only experimentally, hence, here we predict the regulation based on their co-localization with respective operons. We use the name CatR for TFs that regulate only *cat* genes and are co-localized with them; and CatM/BenM for those TFs that control *cat* and/or *ben* genes. This distinction is supported by the branching of the phylogenetic tree of these TFs (Figure 8).

Neither CatR nor CatM/BenM have been found among Rhodocyclales and Comamonadaceae (apart from *Polaromonas naphthalenivorans* CJ2) in our genome set, while most *Burkholderia* spp. and *Ralstonia* spp. have two TFs.

All *Burkholderia* spp. have both CatR and CatM/BenM, where CatR regulates its own gene and the divergently transcribed *catBAC* operon, except for *Burkholderia glumae* BGR1 that has only CatR, which regulates *catBCA* and possibly also adjacent GTPase genes (see below). CatM/BenM seems to regulate its own gene and divergently transcribed *benABCD* in *B. mallei* ATCC 23344, *B. phymatum* STM815, *B. pseudomallei* K96243 and *B. xenovorans* LB400 (Supplementary Figure S4). In those *Burkholderia*, where *catM/benM* is not adjacent to the regulated genes (*B. cepacia* AMMD, *Burkholderia* sp. 383, *B. vietnamiensis* G4), it controls the operon comprised of *cat(B)(C)A–benABCD*, as well as the gene of a COG251 protein (Supplementary Figure S4 and Supplementary Table S2). The possible role of COG251 enamine deaminases (YjgF/RidA family) in the aromatic metabolism was described above in the BoxR section.

*Bordetella petrii* DSM 12804 also has both CatR and CatM/BenM. CatR regulates its own gene and divergently transcribed *catB* and the gene of a COG3181 transport protein. The CatM/BenM regulon is comprised of the TF itself and the divergent operon formed by *catBA*, *benABCD*, the gene of a COG251 protein, *benK*, fragments of *catI* and *catF*, *catDC*, the genes of COG3181, COG1012, COG1062, COG2271 proteins, *benE*, as well as two genes encoding proteins with predicted GTPase activity (Supplementary Figure S5). The fragments of *catI* and *catF* are homologous to *catI* and *catF* of various Comamonadaceae, where they are regulated by an IclR-family TF (Supplementary Table S2). COG1062 and COG1012 dehydrogenases likely convert benzyl-alcohol through benzaldehyde into benzoate, for further degradation by BenABCD. The COG2271 transporter may be involved in import of hydroxybenzoate or some other benzoate-related compound. The GTPases in the regulon are distantly homologous to each other and to the genes forming operons with *catA* in *A. baylyi* ADP1 and *catBCA* in *B. glumae* BGR1.

CatR in *Ralstonia metallidurans* CH34 and both studied *Ralstonia eutropha* strains regulates its own gene and divergently transcribed *catB(C)*–COG3181–(*catD*). Notably, in *R. eutropha* these genes are positioned close to phenol metabolism genes, while in *R. metallidurans* CH34, *catR*, *catM/benM* and their regulated genes form one cluster. CatM/BenM in these three *Ralstonia* species regulates *catA–benABCD* and its own divergent gene, as well as co-transcribed *catB* in *R. eutropha* JMP134, and *catBCD* and COG3246 protein in *R. eutropha* H16 (Supplementary Figure S5). Homologs of the latter in other bacteria are often associated with genes of protocatechuate 3,4-dioxygenase subunits, and other member of this family include 3-keto-5-aminohexanoate cleavage enzyme that catalyzes condensation of the compound with acetyl-CoA yielding 3-aminobutanoyl-CoA and acetoacetate (Bellinzoni et al., 2011). Thus, this COG3246 protein might be a 3-keto acid (e.g., β-ketoadipate) cleavage enzyme that catalyzes carbon-carbon condensation with CoA esters and is involved in the catechol metabolism.

In *R. eutropha* JMP134, CatM/BenM also likely controls the expression of separately localized *benK* and *benE*. Candidate binding sites conforming to the CatR or CatM/BenM consensus have been also observed upstream of plasmid genes encoding a COG3181 protein (*PHG382*) and *catBC*-COG3181 (*PHG405-403*) in *R. eutropha* H16, though they might be regulated by other LysR-family TFs, e.g., MmlR (*PHG389*, see below), PHG401 or PHG406 (Supplementary Table S3).

*Ralstonia pickettii* 12J has only CatM/BenM, which controls its own gene and the divergent *catBCA–benABCDKE* operon. In *Polaromonas naphthalenivorans* CJ2, a similar situationis observed, with *catM/benM* and *benABCDKE* regulated by CatM/BenM (Supplementary Table S2).

### Regulation of Chlorocatechol and 2,4-Dichlorophenoxyacetic Acid Metabolism by ClcR and TfdR/TfdS

In the analyzed bacteria, the *clc* or *tfd* genes of chloroaromatic metabolism have been identified only in *Bordetella petrii* DSM 12804, *Burkholderia xenovorans* LB400, and *Ralstonia eutropha* JMP134 (Figure 8), all of which are prominent in their ability to degrade a remarkable variety of aromatic compounds (Chain et al., 2006; Gross et al., 2008; Lykidis et al., 2010).

The ClcR regulons (Supplementary Table S2) in *B. petrii* and *B. xenovorans* are comprised of *clcR* and the divergently transcribed operon consisting of *clcAB*, the *clcC* gene encoding a COG3181 protein [homologous, but not orthologous to the ones in the CatR(M)/BenM, AphS, AphR, AphT regulons, data not shown], *clcDE*, and possibly also the gene of an AraC-family TF (*Bpet1539*, *Bxe_A1125*). The function of the latter is unknown, but it is distantly homologous to the regulators of anthranilate (AntR, AndR) and benzoate (BenR) degradation, thus it might indeed be involved in the regulation of metabolism of some benzoate derivative.

**FIGURE 9:**
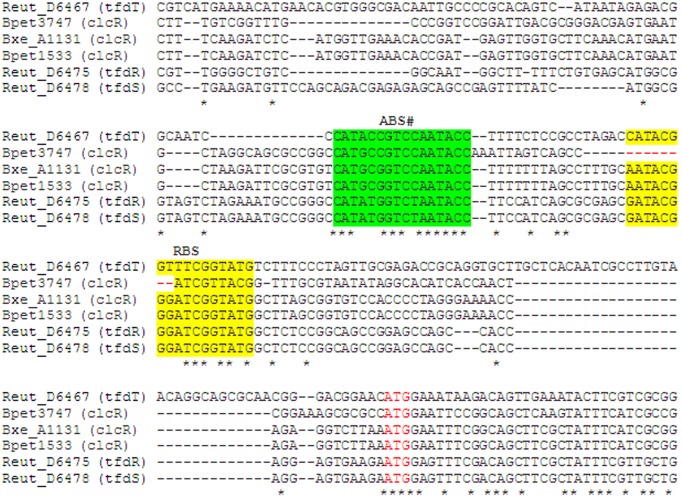
Multiple alignment of the upstream regions of regulatory genes *clcR*, *tfdR*, *tfdS*, *tfdT.* ABS is highlighted with green, RBS – with yellow, start codon ATG is marked with red font. Asterisks mark completely conserved positions of the alignment. #Since genes of TFs are divergent to their regulated genes, ABS sequences here are in reverse complement compared to the sequences (ABS in *catB*, *clcA* upstream regions) described earlier (Parsek et al., 1994; Chugani et al., 1997, 1998; Tover et al., 2000, 2001).

*Bordetella petrii* also has a second copy of the *clc* gene cluster that has the same composition and organization as the first one, except for an AraC-family regulator gene. Notably, this *clc* gene cluster apparently has a specific mode of regulation. It is known that in addition to the AkACC–N_5_–GGTAT motif (Figure 2B), which is the repression binding site (RBS), both CatR and ClcR bind an adjacent activation binding site (ABS) (Parsek et al., 1994; Chugani et al., 1997, 1998; Tover et al., 2000, 2001). A similar ABS was also identified for the *tfd* genes, while the second *clc* gene cluster in *B. petrii* (*Bpet3747*; *Bpet3748*–*3752*) lacks half of the palindromic RBS, yet retains the ABS (Figure 9).

The TfdR/TfdS regulon (Supplementary Tables S2, S3) in *R. eutropha* includes several co-localized, divergently oriented pairs of operons from a plasmid (Leveau and van der Meer, 1996; Laemmli et al., 2000; Vedler et al., 2004):

(i) *tfdT* and *tfdCDEF* (*tfdB* is adjacent, but is not a part of the *tfd* operon). TfdT is highly similar toTfdR/TfdS, but is inactive due to an insertion element;
(ii) *tfdD_II_C_II_E_II_F_II_B_II_K* and *tfdR*, possibly co-transcribed with an adjacent chimerical gene, encoding partially the HTH domain of an IclR-family TF (see below) and partially COG1024 enoyl-CoA hydratase/3-hydroxyacyl-CoA dehydrogenase (*Reut_D6476*). We predict that it may be involved in the conversion of 2,3-didehydroadipyl-CoA to 3-ketoadipyl-CoA;
(iii) *tfdS* and *tfdA*; *tfdS* may form an operon with the gene of an IclR-family TF (*Reut_D6477*) that is distantly homologous to the regulators of the 4-hydroxybenzoate and protocatechuate utilization, PobR and PcaR, and modulates expression of the *tfd* genes (Trefault et al., 2009). The adjacent genes encoding acyl-CoA synthetase COG318, thiolase COG183, and a COG1545 protein are possibly co-transcribed with *tfdA* or, at least, are involved in the lower 2,4-D metabolism. The COG318 protein likely acts on the β-ketoadipate produced by TfdF, while COG183 is likely a β-ketoadipyl-CoA thiolase that further degrades it into acetyl-CoA and succinyl-CoA, thus channeling the 2,4-D metabolism into the TCA cycle. The exact function of the COG1545 protein remains unknown, but it is distantly related to acyl dehydratases and is likely functionally connected to β-ketoadipyl-CoA thiolase, as the genomic context shows that various COG1545 and COG183 proteins are often associated.

### Regulation of 4-Methylmuconolactone Metabolism by MmlR

MmlR and 4-methylmuconolactone metabolism genes were found only in *R. eutropha* H16 and *R. eutropha* JMP134 (Figure 8 and Supplementary Table S2). Notably, in *R. eutropha* H16 these genes are localized on a plasmid, while in *R. eutropha* JMP134 they are chromosomal.

A candidate MmlR binding site has been identified upstream of the *mmlLRFGHIJ* operon based on its conservation in these two species and its high similarity to the binding motifs of the homologous TFs ClcR/TfdR/TfdS and CatR(M)/BenM (Figure 2B, Supplementary Figure S6, and Supplementary Table S3). The similarity of the motifs may result in the cross-regulation by the respective TFs.

### A Novel LysR-Family Regulator of Aromatic Metabolism, CatR^∗^

In five Commamonadaceae species we have identified a TF homologous to CatR, ClcR, and CynR (Figure 8). The phylogenetic footprinting has revealed a possible binding motif (Figure 2B), an odd palindrome conforming to the general LysR-family consensus (T–N_11_–A). Similarly to CatR, this TF is predicted to regulate diverse combinations of genes (Supplementary Figure S7) encoding CatA, CatB, CatC homologs and COG3181 proteins (all of them form branches on the phylogenetic trees that are separate from those controlled by CatR, data not shown; Supplementary Table S2), and its own divergently transcribed gene (except in *Delftia acidovorans* SPH-1). Notably, all studied bacteria that have this TF lack CatR(M)/BenM, except for *Polaromonas naphthalenivorans* CJ2, where both these regulators belong to the same gene cluster, with *ben* genes under the CatM/BenM control. Thus, this TF either substitutes CatR(M)/BenM, or shares functions with it.

## Discussion

This comparative study showed complexity and variability in the regulation of aromatic metabolism in Betaproteobacteria. The aromatic metabolism is organized by key substrates, which are the merging points of various *upper* metabolic pathways and are further degraded by the *lower* pathways flowing into the central metabolism. The *upper* parts of pathways, rare pathway variants, and degradative pathways of exotic and complex, in particular, xenobiotic compounds are generally controlled by a single TF each (e.g., the *bph*, *box, mml*, or *tfd* genes). The regulation of more common and/or central parts of the metabolism (e.g., the *aph, ben*, or *cat* genes) may vary even between close species and could involve several TFs with diverse modes of interaction (Figure 1 and Supplementary Table S2). In particular, TFs may simultaneously control certain operons (dual regulation), the regulation of a pathway may be divided between different TFs (shared regulation), or a TF may control expression of another TF (cascade regulation). For example, in *Acidovorax* sp. JS42 and *L. cholodnii* SP-6, the *aph* genes are under shared and/or dual regulation by AphS, AphR and AphT (Figure 4), and in *D. aromatica* RCB and *Thauera* sp. MZ1T there is cascade regulation of BenR by AphS (Figure 5).

These observations are likely not restricted to Betaproteobacteria and can be propagated to other classes, e.g., Gammaproteobacteria. Most aromatic metabolism pathways and respective TFs studied here have been identified in Gammaproteobacteria as well, and are similarly regulated. Some examples are *ben* and *cat* genes regulated by homologous TFs BenM and CatM, respectively, in *Acinetobacter baylyi* ADP1, and *ben* genes regulated by BenR in *Pseudomonas putida* (Collier et al., 1998; Cowles et al., 2000; Ezezika et al., 2006; Li et al., 2010). An interesting direction of research could be the analysis of horizontal gene transfer between and within Beta- and Gammaproteobacteria involving both pathway operons and respective TFs. One specific question could be whether operons always travel with their regulators, or there are cases when a newly arrived operon falls under regulation by a pre-existing TF. As more genomes become available, one might hope to reconstruct the evolutionary history of the metabolic genes and their regulators in considerable detail.

**FIGURE 10:**
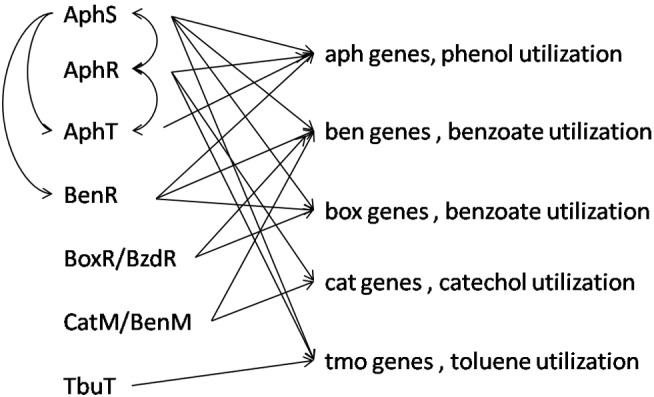
Integrated scheme of interactions between aromatic metabolism TFs and regulated genes in all studied genomes.

Among the studied genomes, *D. aromatica* RCB and *Thauera* sp. MZ1T have the most complex interaction of studied aromatic regulatory proteins, and the most frequent and, at the same time, variable connection is between AphS, AphR, AphT, and BenR (Figure 10 and Supplementary Table S2).

We have identified a novel LysR-family TF, CatR^∗^, that regulates the metabolism of catechol (or some catechol derivative) and either substitutes CatR(M)/BenM, or shares functions with it.

We have also predicted several new members of aromatic regulons, with some COGs belonging to several regulons (Table 1), the most notable example being COG3181 transporter proteins: in different species, genes from this COG have been assigned to AphS, AphR, AphT, ClcR, CatR(M)/BenM, and CatR^∗^ regulons. Validation of predicted metabolic functions may be an object of further experimental study.

**Table 1:** Predicted functions of the main new members of aromatic regulons.

COG or other ID	Predicted function	Regulated by TFs
COG115	4-Amino-4-deoxychorismate lyase	AphR
COG183	β-ketoadipyl -CoA thiolase	TfdR(S)
COG251	Enamine deaminase involved in utilization of benzoate derivative and stress resistance	CatM/BenM, BoxR
COG318	β-ketoadipyl -CoA synthetase, involved in the lower 2,4-D metabolism	TfdR(S)
COG1012	Benzaldehyde dehydrogenase	CatM/BenM
COG1024	Enoyl-CoA hydratase/3-hydroxyacyl-CoA dehydrogenase, possibly involved in the conversion of 2,3-didehydroadipyl-CoA to 3-ketoadipyl-CoA	TfdR(S)
COG1024/ COG447	3-Hydroxyacyl-CoA hydro-lyase	BoxR
COG1062	Benzyl-alcohol dehydrogenase	CatM/BenM
COG1250	3-Hydroxyadipyl-CoA dehydrogenase	BoxR
COG1545	Distantly related to acyl dehydratases, functionally connected to β-ketoadipyl-CoA thiolase	TfdR(S)
COG1894, COG3383	Formate dehydrogenase possibly involved in the oxidation of formate generated by BoxC	BoxR
COG2271	Hydroxybenzoate or some benzoate-related compound transporter	CatM/BenM
COG3181	Aromatic transporter family receptor	AphS, AphR, AphT, ClcR, CatR(M)/BenM, CatR^∗^
COG3246	3-Keto acid (possibly, β-ketoadipate) cleavage enzyme involved in the catechol metabolism	CatM/BenM
COG3485	Dioxygenase of some catechol derivative, possibly 3,4-dihydroxybenzoate or 3,4-dihydroxyphenylacetate	AphT
COG3945	Protein with hemerythrin/HHE cation-binding motif, possibly protecting the cell from toxic compounds and/or acting as an oxygen store/scavenger	AphS, BenR, BoxR
COG3971	2-Oxo-hepta-3-ene-1,7-dioic acid hydratase	AphT
COG4231	2-Ketoacid ferredoxin oxidoreductase, distantly homologous to arylpyruvate ferredoxin oxidoreductases	AphS, BenR
COG4447, COG1033, PF07044, DUF1302	Proteins possibly involved in coping with toxic concentrations of aromatic compounds and with the solvent-stress response	AphS, AphR, AphT


In addition to the structure of regulatory networks and regulon content, we have also made some observations about the possible mechanism of regulation. In particular, AphR belongs to the XylR/DmpR group of regulators, which also includes TbuT, HbpR, and other TFs that control metabolism of aromatic compounds (Arai et al., 1999; Jaspers et al., 2001; Tropel and van der Meer, 2002). These TFs are known to bind pairs of palindromic sequences, approximately 16–20 nt in length and with a 29–42 nt distance between their centers, which are usually localized 100–200 nt upstream of the –12/–24 target promoters (Jaspers et al., 2001; Tropel and van der Meer, 2002). The correct spacing between two binding sites is necessary for the cooperative binding shown for XylR/DmpR TFs: both sites should face the same side of the DNA helix, e.g., the centers of binding sites are separated by three complete DNA helical turns in the case of XylR and DmpR, and by three or four turns, in the case of HbpR (Jaspers et al., 2001; Tropel and van der Meer, 2002). We have observed that almost all genes predicted to be under regulation of AphR and its homologs in the studied bacteria are preceded by multiple sites, separated by three (or rarely by one) DNA turns (Supplementary Table S3).

Our study also supports previous observations that genes of aromatic metabolism are often organized in very long supraoperonic clusters and are associated with mobile DNA elements that facilitate their transfer between genomes. For example, transposases and/or integrases flank the *aph* genes in *B. vietnamiensis* G4 and *M. petroleiphilum* PM1; *bph*, *box*, and *clc* genes in *B. xenovorans* LB400; *cat* and *clc* genes in *B. petrii* DSM 12804, etc (Figure 3, 4, 6 and Supplementary Figure S5). These features allow for biochemical diversity of bacteria and their rapid adaptation for various compounds, including xenobiotics.

## Data Availability

All datasets generated for this study are included in the manuscript and/or the Supplementary Files.

## Author Contributions

IS and MG conceived the study. IS designed and performed the comparative analysis, and visualized results. IS and MG participated in writing and revising the manuscript, read and approved the submitted version.

## Conflict of Interest Statement

The authors declare that the research was conducted in the absence of any commercial or financial relationships that could be construed as a potential conflict of interest.
