# Supplementary material for: Comparative Genomic Analysis of the Regulation of Aromatic Metabolism in Betaproteobacteria
Source: Front Microbiol. 2019 Mar 29;10:642. doi: 10.3389/fmicb.2019.00642 (PMC6449761; doi:10.3389/fmicb.2019.00642)
Supplement: Supplementary file 1 [file Data_Sheet_1.docx]

**
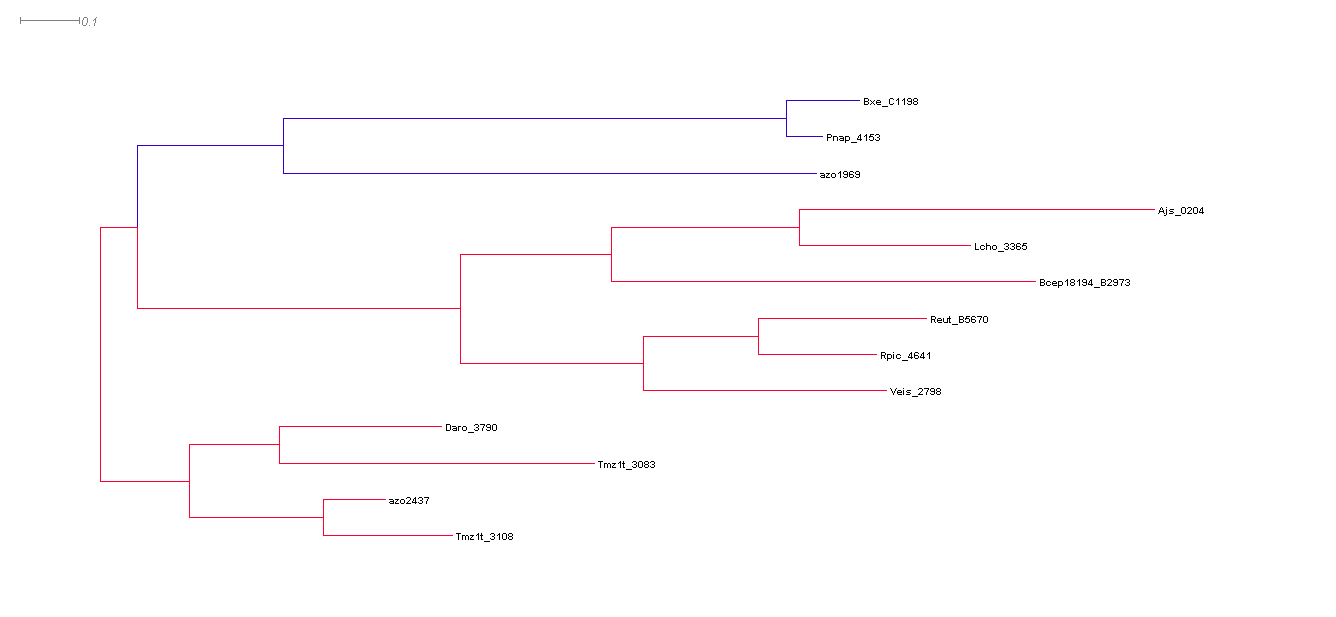
**

**Figure S1. Phylogenetic tree of AphS and BphS regulators.**

AphS shown with red, BphS – blue


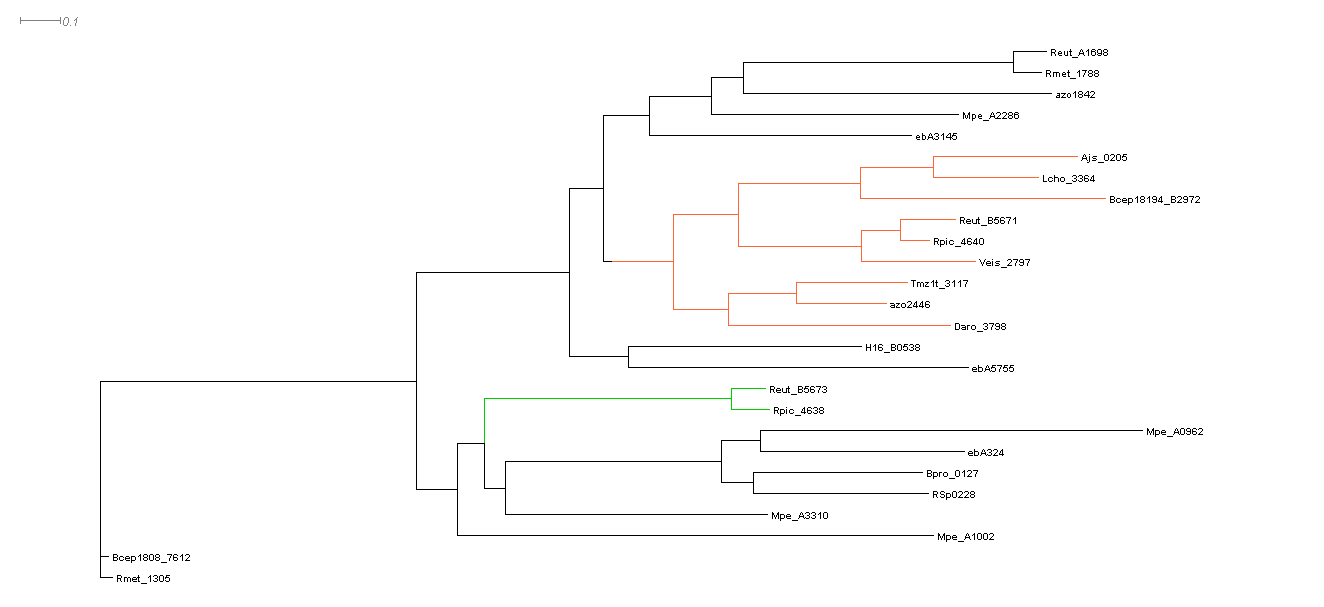


**Figure S2. Phylogenetic tree of AphR, TbuT regulators and their close homologs.**

AphR shown with red, TbuT – green


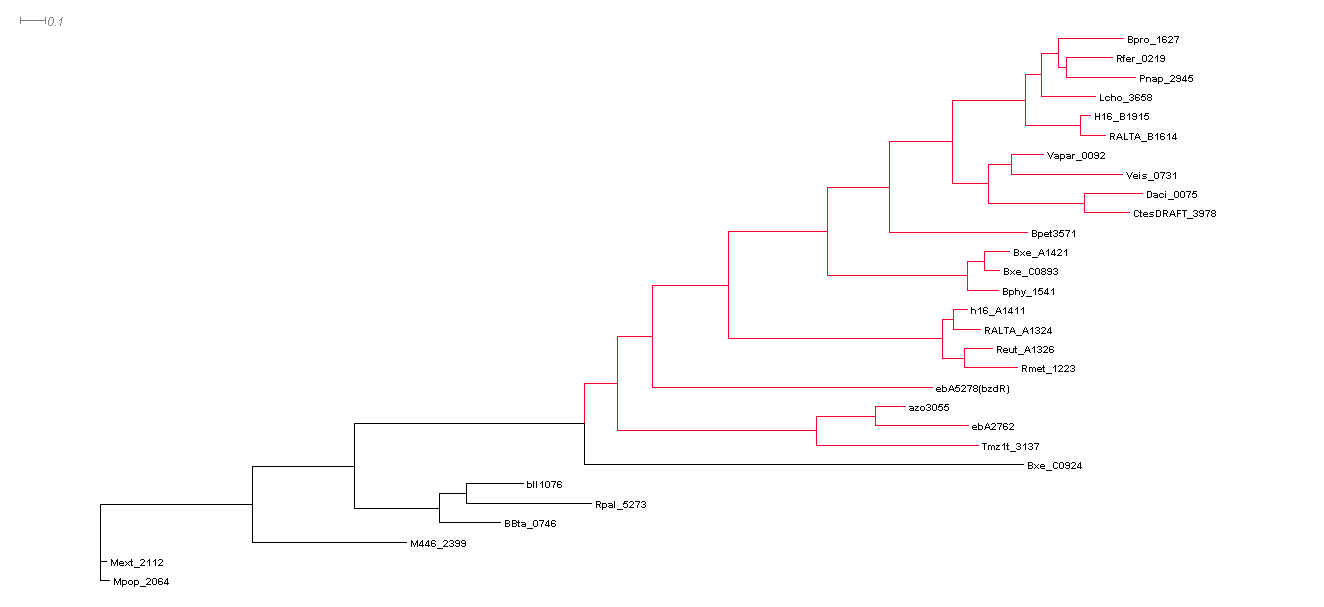


**Figure S3. Phylogenetic tree of BoxR and BzdR regulators**

BoxR/BzdR shown with red, outgroup – black


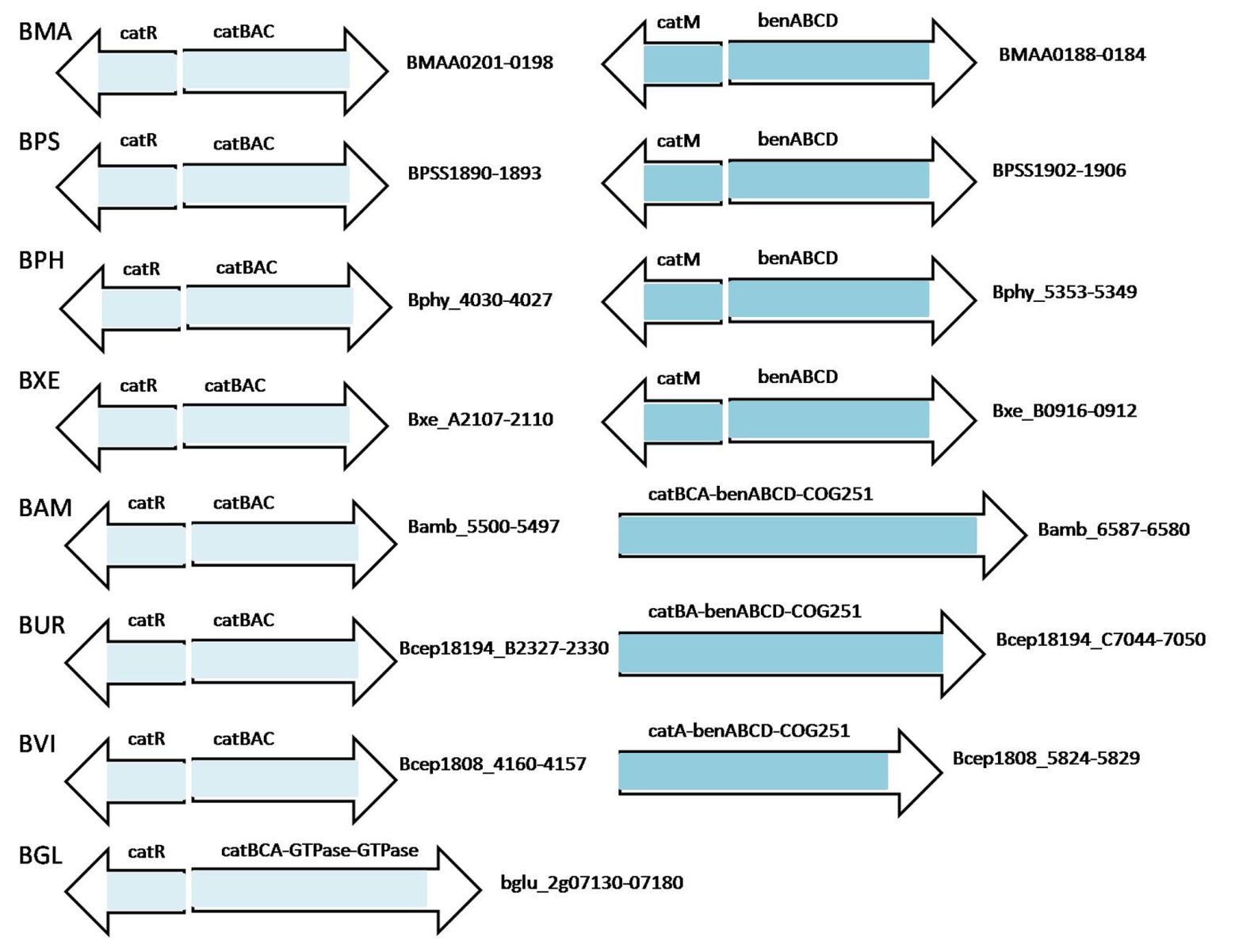


**Figure S4. Operon organization and regulation of aromatic metabolism genes regulated by CatR and CatM/BenM in *Burkholderia* spp.**

Color denotes regulation by the following TFs: CatR – light blue, CatM/BenM – blue. Genes/operons are shown by arrows. Genome abbreviations as in Suppl. Table S2.


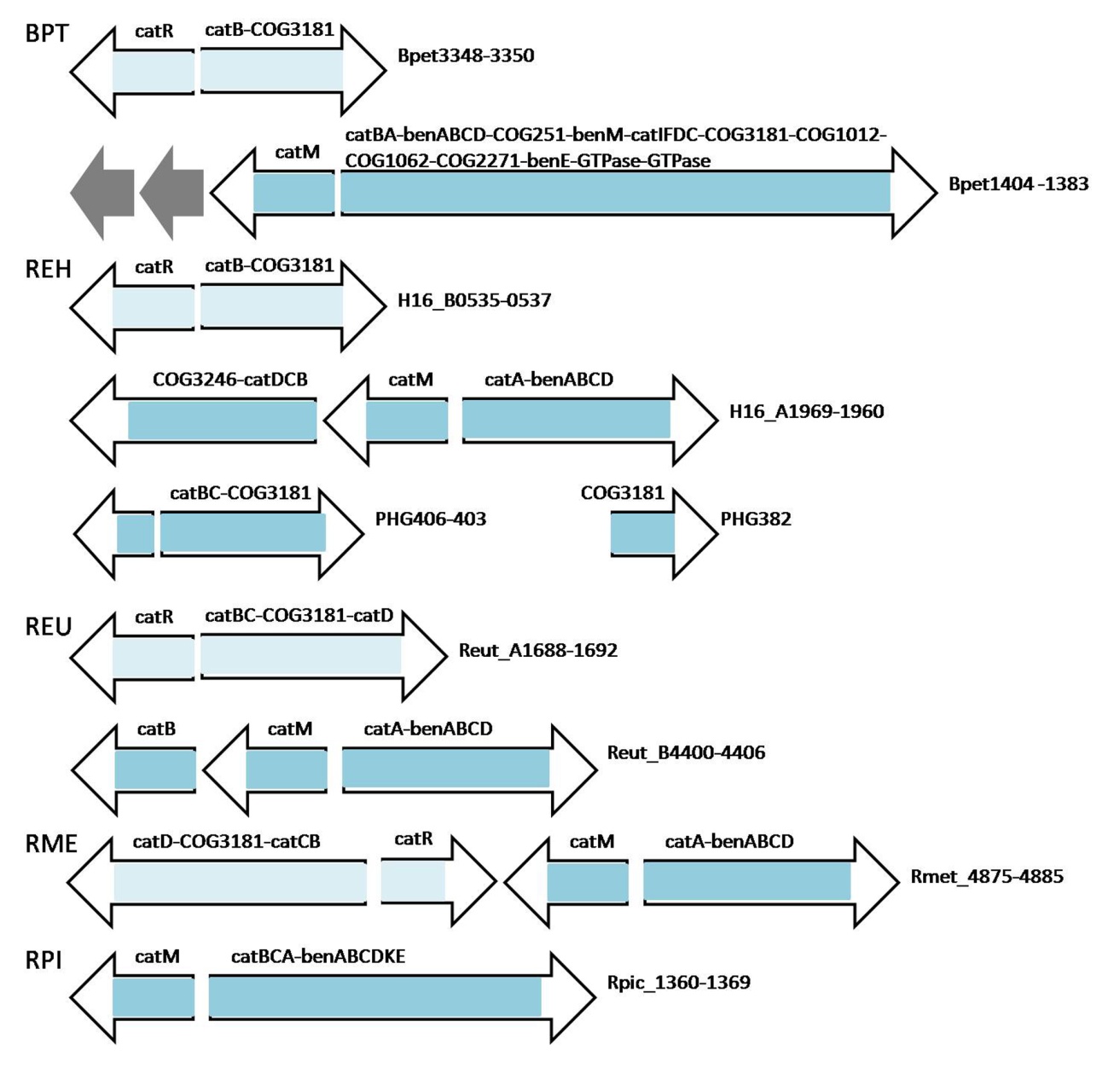


**Figure S5. Operon organization and regulation of aromatic metabolism genes regulated by CatR and CatM/BenM in *B. petrii* DSM 12804 and *Ralstonia* spp.**

Color denotes regulation by the following TFs: CatR – light blue, CatM/BenM – blue. Genes/operons are shown by arrows, grey arrows denote transposases. Genome abbreviations as in Suppl. Table S2.

BPSS1903 (benA) TGCAGGCGCTCGGCCG---CGCGCGTGAAGTTCTGCTCCTCCGCGACCGCGACG---AAG

BMAA0187 (benA) TGCAGGCGCTCGGCCG---CGCGCGTGAAGTTCTGCTCCTCCGCGACCGCGACG---AAG

Reut_A1508 (mmlL) -GCTGGCGCGACGCTGGCCGGCGTACGAAGAC--------------ATCCGACGCGAATC

PHG390 (mmlL) -------------------AACCCAGGAGGTT--------------TCACGACG------

* ** * *****

BPSS1903 (benA) TAGCGTAGATGCCGCAGTTCCATCTTCATACCTCGAGGATATCGATCCGATACTCAATCA

BMAA0187 (benA) TAGCGTAGATGCCGCAGTTCCATCTTCATACCTCGAGGATATCGATCCGATACTCAATCA

Reut_A1508 (mmlL) TATGCTCGACTCCACCAA------CCCATACCTGACAGGTATGGGTTGATGACATCATTG

PHG390 (mmlL) TATTGTTGATT-CGCCAAGGCGCTGCGATACCCTGTAGATATCGAATCCA-ACATTTTCA

** * ** * * ***** * *** * ** *

BPSS1903 (benA) GTGTTGGACGCACCGTTTTGCCGGCAACTATCCTC-------AATCTGACCG--------

BMAA0187 (benA) GTGTTGGACGCACCGTTTTGCCGGCAACTATCCTC-------AATCTGACCG--------

Reut_A1508 (mmlL) GTCTTGGACGT-TCGAGCCGCCGGGGTCCATACTTTTCTCCCTGTCTGGCCG--------

PHG390 (mmlL) GTCTTGGACACCCCAAGAC-TTGTAACTCATACTCTGACATACATGCGAGTGTGAAATGC

** ****** * * ** ** * * *

BPSS1903 (benA) --------------------------CGCTGTCGTCGAGCACTATACCTGCGAAGCATCG

BMAA0187 (benA) --------------------------CGCTGTCGTCGAGCACTATACCTGCGAAGCATCG

Reut_A1508 (mmlL) -----GCGCATAAGGCCTCGTGAAAGCCCCCTTTCGGATTGGAAAATTTGCGGGGC-TCG

PHG390 (mmlL) ACCGCACGTGTGAATCTGCCTGAAACCCCTCTTTTGGAATGCAAAATTGGCGGCGGGATG

* * * ** * * *** * *

BPSS1903 (benA) CAGGTTCACCCGGGGTTTCCCATGCGACGCGCGGCGCGGCGCCTCGCCGGACCGCGC---

BMAA0187 (benA) CAGGTTCACCCGGGGTTTCCCATGCGACGCGCGGCGCGGCGCCTCGCCGGACCGCGC---

Reut_A1508 (mmlL) AAAATTCACCGA-----TCCTTTACGCCGACAACCGC------------TACCACGACCC

PHG390 (mmlL) AAGA-TCACCGA-----TGCTTGACGCCGGCCAATGCGGGCCTTGGCCATACCGCAA---

* ***** * * ** ** ** *** *

BPSS1903 (benA) -------GTCCGCACGCGCGCCGCC-CCCGATCAGAGAGCCCATCAGGAGACGC-CCCC

BMAA0187 (benA) -------GTCCGCACGCGCGCCGCC-CCCGATCAGAGAGCCCATCAGGAGACGC-CCCC

Reut_A1508 (mmlL) GCGAGTGCTGCGGGCGCTGGCAGCAGCACCATCGGACAAT------GGAGACAAGGCAC

PHG390 (mmlL) -AGACAGGTCCCAAGGCGGGCCATCGCAACACCAGAAAA-------GGAGACACGGCAC

* * ** ** * * * ** * ****** * *

**Figure S6. Multiple alignment of the upstream regions of *mmlL* and *benA* genes**

Upstream regions are -300 to 0 nt relative to the gene start. Similar binding sites of CatM/BenM (*benA* upstreams) and MmlR (*mmlL* upstreams) are highlighted with yellow.


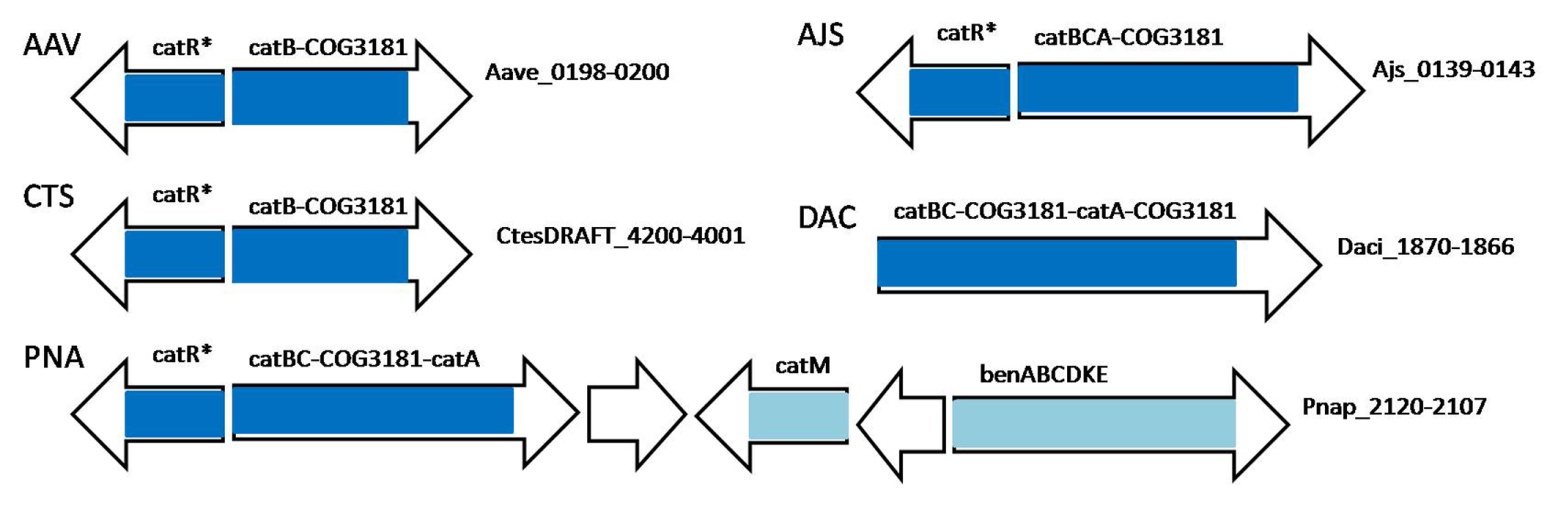


**Figure S7. Operon organization and regulation of aromatic metabolism genes regulated by CatR*.**

Color denotes regulation by the following TFs: CatM/BenM – light blue, CatR* – dark blue. Genes/operons are shown by arrows. Genome abbreviations as in Suppl. Table S2.
