# Supplementary material for: Comparative Genomic Analysis of the Regulation of Aromatic Metabolism in Betaproteobacteria
Source: Front Microbiol. 2019 Mar 29;10:642. doi: 10.3389/fmicb.2019.00642 (PMC6449761; doi:10.3389/fmicb.2019.00642)
Supplement: Supplementary file 2 [file Table_1.DOCX]

**Table S1. Main proteins of phenol, biphenyl, benzoate, and (chloro/methyl)catechol utilization.**

| AphB, COG2514 | catechol-2,3-dioxygenase (related to DmpB, LapB, XylE) |
| --- | --- |
| AphC, COG1012 (PutA) | 2-hydroxymuconic semialdehyde dehydrogenase (related to DmpC, LapC, XylG) |
| AphE/BphE(H), COG3971 | 2-hydroxypenta-2,4-dienoate hydratase (related to MhpD, DmpE, LapE, XylJ) |
| AphF/BphG(J), COG4569 (MhpF) | acetaldehyde dehydrogenase, acylating (related to MhpF, DmpF, LapF, XylQ) |
| AphG/BphF(I), COG119 (LeuA) | 4-hydroxy-2-ketovalerate aldolase (related to MhpE, DmpG, LapG, XylK) |
| AphH, COG3971 | 4-oxalocrotonate decarboxylase (related to DmpH, LapH, XylI) |
| AphI,COG1942 | 4-oxalocrotonate tautomerase (related to DmpI, XylH) |
| AphJ, COG3181 | putative transport protein involved in phenol metabolism |
| AphK, PF06099 | phenol hydroxylase subunit (related to DmpK, LapK, PoxA) |
| AphL, PF02332 | phenol hydroxylase subunit (related to DmpL, LapL, PoxB) |
| AphM, PF02406 | phenol hydroxylase subunit (related to DmpM, LapM, PoxC) |
| AphN, PF02332 | phenol hydroxylase subunit (related to DmpN, LapN, PoxD) |
| AphO, PF04663 | phenol hydroxylase subunit (related to DmpO, LapO, PoxE) |
| AphP, COG2871 (NqrF) | phenol hydroxylase ferredoxin oxidoreductase subunit (related to DmpP, LapP, PoxF) |
| AphQ, COG633 (Fdx) | phenol hydroxylase ferredoxin subunit (related to LapQ, PoxG, XylT) |
| AphR, COG2204 (AtoC)/COG3604 (FhlA)/COG3829(RocR) | sigma54-specific activator of phenol metabolism |
| AphS, COG1802 (GntR) | GntR-family repressor of phenol metabolism |
| BphA1, COG4638 (HcaE) | biphenyl 2,3-dioxygenase alpha subunit |
| BphA2, COG5517 | biphenyl 2,3-dioxygenase small beta subunit |
| BphA3, COG2146 (NirD) | biphenyl 2,3-dioxygenase ferredoxin subunit |
| BphA4, COG1251 (NirB) | biphenyl 2,3-dioxygenase ferredoxin reductase subunit |
| BphB, COG1028 (FabG) | 2,3-dihydrobiphenyl-2,3-diol dehydrogenase |
| BphC, PF00903 | 2,3-dihydroxybiphenyl-1,2-dioxygenase |
| BphD, COG596 (MhpC) | 2-hydroxy-6-oxo-6-phenylhexa-2,4-dienoate hydrolase |
| BphK, COG625 (Gst) | glutathione S-transferase |
| BphS, COG1802 (GntR) | GntR-family repressor of biphenyl metabolism |
| BphX, PF06139 | membrane protein, biphenyl utilization pathway |
| BenA, COG4638 (HcaE) | benzoate-1,2-dioxygenase large alpha subunit |
| BenB, COG5517 | benzoate-1,2-dioxygenase small beta subunit |
| BenC, COG2871 (NqrF) | benzoate-1,2-dioxygenase electron transfer ferredoxin oxidoreductase subunit |
| BenD, COG1028 (FabG) | 1,6-dihydroxycyclohexa-2,4-diene-1-carboxylate dehydrogenase |
| BenK, COG2271 (UhpC)/COG2223 (NarK) | benzoate transporter, major facilitator superfamily MFS_1 |
| BenE, COG3135 (BenE) | benzoate membrane transport protein |
| BenR, COG2207 (AraC) | AraC-family regulator of benzoate utilization |
| BoxA, COG1143 (NuoI)/ COG1146 | benzoyl-CoA oxygenase/reductase component A |
| BoxB, COG3396 | benzoyl-CoA oxygenase (2,3-epoxidase) component B |
| BoxC, COG1024 (CaiD) | 2,3-epoxybenzoyl-CoA dihydrolase |
| BoxD (BoxZ), COG1012 (PutA) | 3,4-dehydroadipyl-CoA semialdehyde dehydrogenase |
| BoxR, COG703 (AroK) | XRE-family regulator of aerobic epoxide box-pathway benzoate metabolism |
| BzdA/BclA, COG365 (Acs)/COG318 (CaiC) | ATP-dependent benzoate-CoA ligase |
| BzdM/BadB/Fdx, COG1142 (HycB) | benzoyl-CoA reductase associated ferredoxin |
| BzdN/BcrC/BadD, COG1775 (HgdB) | benzoyl-CoA reductase gamma subunit |
| BzdO/BcrB/BadE, COG1775 (HgdB) | benzoyl-CoA reductase beta subunit |
| BzdP/BcrD/BadF, COG1924 | benzoyl-CoA reductase delta subunit |
| BzdQ/BcrA/BadG, COG1924 | benzoyl-CoA reductase alpha subunit |
| BzdR, COG703 (AroK) | XRE-family regulator of anaerobic benzoate metabolism |
| BzdS | hypothetical protein |
| BzdT, COG456(RimI) | N-acetyltransferase, putative regulatory protein |
| BzdU, COG613 | metal-dependent phosphoesterase (PHP family) |
| BzdV, COG493 (GltD) | NADPH-dependent oxidoreductase possibly involved in benzoyl-CoA reductase associated ferredoxin reduction |
| BzdW/BadK/Dch, COG1024 (CaiD) | cyclohexa-1,5-diene-1-carbonyl-CoA/cyclohexa-1-ene-carbonyl-CoA hydratase |
| BzdX/BadH/Had, COG1064 (AdhP) | 2-hydroxycyclohexane-1-carbonyl-CoA/6-hydroxycyclohex-1-ene-1-carbonyl-CoA dehydrogenase |
| BzdY/BadI/Oah, COG447 (MenB) | 2-ketocyclohexane-1-carbonyl-CoA/6-ketocyclohex-1-ene-1-carbonyl-CoA hydrolase |
| BzdZ, COG1028 (FabG) | 3-ketoacyl-[acyl-carrier-protein] reductase |
| CatA, COG3485 (PcaH) | catechol-1,2-dioxygenase |
| CatB, COG4948 | muconate cycloisomerase, lactonizing enzyme |
| CatC, COG4829 (CatC1) | muconolactone delta-isomerase |
| CatD/PcaD, COG596 (MhpC) | 3-ketoadipate enol-lactonase |
| CatF/PcaF, COG183 (PaaJ) | 3-ketoadipyl-CoA thiolase |
| CatI/PcaI, COG1788 (AtoD) | 3-ketoadipate CoA-transferase alpha subunit |
| CatJ/PcaJ, COG2057 (AtoA) | 3-ketoadipate CoA-transferase beta subunit |
| CatM/BenM, COG583 (LysR) | LysR-family regulator of catechol and benzoate utilization |
| CatR, COG583 (LysR) | LysR-family regulator of catechol utilization |
| ClcA, COG3485 (PcaH) | 3-chlorocatechol-1,2-dioxygenase |
| ClcB, COG4948 | chloromuconate cycloisomerase, lactonizing enzyme |
| ClcC, COG3181 | putative transport protein involved in chloroaromatic metabolism |
| ClcD, COG412 | dienelactone hydrolase |
| ClcE, COG1454 (EutG) | maleylacetate reductase |
| ClcR, COG583 (LysR) | LysR-family regulator of 3-chlorocatechol utilization |
| MmlF, COG1788 (AtoD) | 4-methyl-3-ketoadipyl-CoA transferase, alpha subunit |
| MmlG, COG2057 (AtoA) | 4-methyl-3-ketoadipyl-CoA transferase, beta subunit |
| MmlH, COG2271 (UhpC) | transporter of extracellular muconolactones or dicarboxylic acids |
| MmlI, PF09448 | 4-methylmuconolactone methylisomerase |
| MmlJ, COG4829 (CatC1) | methylmuconolactone isomerase |
| MmlL, COG491 (GloB) | metal-dependent 4-methyl-3-ketoadipate enol-lactone hydrolase |
| MmlR, COG583 (LysR) | LysR-family regulator of 4-methylmuconolactone metabolism |
| TfdA, COG2175 (TauD) | 2,4-dichlorophenoxyacetate/alpha-ketoglutarate dioxygenase |
| TfdB(I/II), COG654 (UbiH) | 2,4-dichlorophenol 6-monooxygenase/hydroxylase |
| TfdC(I/II), COG3485 (PcaH) | 3,5-dichlorocatechol 1,2-dioxygenase |
| TfdD(I/II), COG4948 | 2,4-dichloromuconate cycloisomerase |
| TfdE(I/II), COG412 | 2-chlorodienelactone hydrolase |
| TfdF(I/II), COG1454 (EutG) | (2-chloro)maleylacetatereductase |
| TfdK, COG2271 (UhpC) | 2,4-dichlorophenoxyacetate transporter |
| TfdR/TfdS, COG583 (LysR) | LysR-family regulator of 2,4-dichlorophenoxyacetate utilization |
| TfdT, COG583 (LysR) | LysR-family regulator of 2,4-dichlorophenoxyacetate utilization, inactivated |
| TmoA, PF02332 | toluene-4-monooxygenase subunit A |
| TmoB/TbuU, PF06234 | toluene-4-monooxygenase subunit B |
| TmoC/TbuB, COG2146 (NirD) | toluene-4-monooxygenase ferredoxin subunit C |
| TmoD/TbuV, PF02406 | toluene-4-monooxygenase subunit D |
| TmoE/TbuA, PF02332 | toluene-4-monooxygenase subunit E |
| TmoF/TbuC, COG2871 (NqrF) | toluene-4-monooxygenase ferredoxin oxidoreductase subunit |
| TbuT, COG3604 (FhlA) | Fis-type sigma54-specific regulator of toluene metabolism |
| TbuX, COG2067 (FadL) | outer membrane protein, involved in toluene uptake and degradation (homologous to TodX, XylN) |
